# Mixed-methods process evaluation of the EACH-B intervention in UK secondary schools: Delivery fidelity, stakeholder responses and contextual influences

**DOI:** 10.1136/bmjph-2024-002491

**Published:** 2025-10-21

**Authors:** Sarah Jenner, Mary Barker, Sofia Strömmer, Sarah Shaw, Laila Khawaja, Millie Barrett, Kathryn Woods-Townsend, Donna Lovelock, Lisa Bagust, Naomi Leonard, Wendy Lawrence, Danielle Lambrick, Judit Varkonyi-Sepp, Hamid Homatash, Patricia Coakley, Christina Vogel, Leanne Morrison, Mary Christina Horsfall, Hazel Inskip, Janis Baird

**Affiliations:** 1 MRC Lifecourse Epidemiology Centre, Southampton, UK; 2 University of Southampton School of Psychology, Southampton, UK; 3 University of Southampton School of Health Sciences, Southampton, UK; 4 NIHR Southampton Biomedical Research Centre, Southampton, UK; 5 University of Southampton School of Healthcare Enterprise and Innovation, Southampton, UK; 6 University of Southampton School of Primary Care Population Science and Medical Education, Southampton, UK; 7 School of Science and Engineering, Glasgow Caledonian University, Glasgow, UK; 8 Department of Public Health Primary Care and Food Policy, City St George's, University of London, London, UK

**Keywords:** Adolescent, Education, Public Health, Qualitative Research

## Abstract

**Background:**

The Engaging Adolescents in Changing Behaviour trial tested the impact of an adolescent diet and physical activity intervention involving: teacher training in Healthy Conversation Skills, a health education module (LifeLab) and a gamified smartphone application (‘the app’). The process evaluation described in this paper examined the implementation, context and mechanisms of impact of the intervention (the core elements of the Medical Research Council guidance on process evaluation of complex interventions), to identify how and why participants did or did not engage with each element of the trial and the intervention.

**Methods:**

A mixed-methods approach was employed. 51 interviews with students, teachers and parents from 11 secondary schools were conducted and analysed using thematic analysis. Quantitative data reported numbers of students who attended LifeLab and downloaded and used the app.

**Results:**

87.9% of eligible students attended LifeLab and 45.7% of eligible students downloaded the app. Students (n=72) and teachers (n=16) had positive experiences of LifeLab and the teacher training, despite the pandemic preventing in-person delivery for some schools. Students engaged in a limited way with the app. Parents (n=11) had positive views of the research but little knowledge of the intervention. Students valued learning about their own health, and teachers were enthusiastic about supporting their students’ health but struggled to find opportunities to do so.

**Conclusions:**

Schools are appropriate settings in which to implement health interventions, but the research must benefit teachers and students. Most students did not engage with the app because they were not presented with it in a way that motivated them to use it. Careful consideration of the design of apps is required to encourage students to use them. Parents should be involved in research in ways that are accessible to them.

**WHAT IS ALREADY KNOWN ON THIS TOPIC**

- In line with the developmental origins of health and disease theory, adolescence is a life stage during which intervention to improve health can be highly beneficial for both current and future generations.
- The most effective intervention strategies to support young people to change their health behaviours are yet to be identified.

**WHAT THIS STUDY ADDS**

- The out-of-school, experiential learning provided by LifeLab—one element of the Engaging Adolescents in Changing Behaviour intervention—was effective in engaging students and teachers in evidence-based health education.
- However, the smartphone application element of the intervention was not able to capture young people’s attention or support them to achieve their health goals.

**HOW THIS STUDY MIGHT AFFECT RESEARCH, PRACTICE OR POLICY**

- This study highlights the importance of schools as effective settings for health interventions, combining researchers’ expertise with teachers’ understanding of school systems.
- Effective interventions will likely use in-school and experiential learning, engaging app designs and collaborative approaches involving schools, parents and researchers to support adolescents to adopt healthier behaviours.

## Introduction

 Adolescents are the parents of the next generation and establishing good preconception health in both girls and boys is known to influence the health of their offspring.^1^ Current trends show that UK adolescents have the worst diets and are the least physically active of any age group.^2^ Just 8% of UK adolescents meet nutritional guidelines recommending that individuals consume five portions of fruits or vegetables every day,^3^ and one-third are overweight or obese.^4^ Data from the Sport England ‘Active Lives’ survey also showed that less than 50% of young people aged 5–16 years in the UK met physical activity guidelines recommended by the Chief Medical Officer in 2023–2024.^5^

Therefore, developing effective interventions to improve adolescent health is essential. Certain features of behavioural interventions are more likely to be effective during adolescence, including those which actively engage adolescents in behaviour change that aligns with their values.^6 7^ Strategies such as codesign, personalisation, use of adolescents’ social networks and collaboration with parents, communities, schools and youth agencies have also been shown to be effective.^8^

Funded by the National Institute for Health Research PGfAR in 2017, the EACH-B (Engaging Adolescents in Changing Behaviour) programme aimed to develop and test a multicomponent intervention to improve diet and physical activity in adolescents (trial registration ISRCTN 74109264).^9^ EACH-B was designed using a person-based approach,^10^ following extensive engagement with adolescents, parents, schools, youth groups and other stakeholders. The person-based approach advocates the use of ‘guiding principles’, which were developed in the early phases of EACH-B and which informed the development of the intervention alongside extensive participant and public engagement. The person-based approach aims to improve the likelihood of interventions being able to effectively improve target outcomes, though they cannot guarantee that every intervention will be successful in achieving sustained behaviour change.

**Figure 1:**
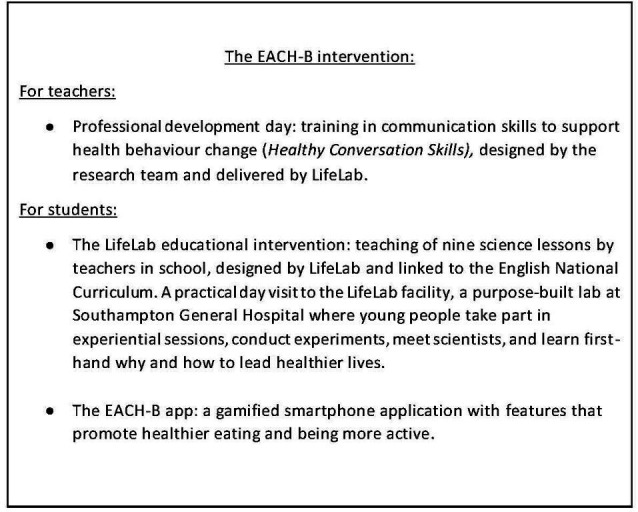
The three elements of the EACH-B intervention. EACH-B, Engaging Adolescents in Changing Behaviour.

The intervention (see figure 1) consisted of three linked elements: Healthy Conversation Skills training,^11^ the LifeLab educational intervention and a gamified smartphone application (henceforth referred to as ‘the app’). The design and content of the app were mapped on to 27 behaviour change techniques (BCTs)^12^ derived from initial qualitative work that was conducted in the development phase of the intervention. A detailed description of these BCTs is available in the online supplemental document 1 The app development was also based on published evidence suggesting that digital interventions for adolescents, when combined with BCTs, can be effective in improving health behaviours.^13^ Rose *et al* conducted a systematic review of digital health interventions to improve diet and physical activity behaviours in adolescents and concluded that further study on the specific effects of smartphone app-based interventions is required in order to adequately assess the efficacy of apps as intervention components.^13^

The effectiveness of the EACH-B intervention was assessed using a cluster-randomised controlled trial with coprimary outcomes of changes in diet quality and total daily physical activity (measured using a 20-item Food Frequency Questionnaire^14^ and GENEActiv^15^ accelerometers, respectively) in secondary school students aged 12 to 14 years between 2021 and 2023 (publication forthcoming). An extensive process evaluation was also conducted alongside the trial to understand how, under what circumstances and for whom the intervention worked.

Medical Research Council (MRC) guidance recommends that process evaluations of complex interventions consider intervention implementation, mechanisms of impact and context.^16^ Implementation captures fidelity (whether the intervention was delivered as intended), dose (the quantity of intervention) and reach (whether the intervention reached the intended audience). Assessing mechanisms of impact involves examining how the intervention produced change and enables replication of effects in other settings. Context relates to factors external to the intervention such as policy or practice changes that might have affected intervention delivery. This paper reports the findings of the EACH-B process evaluation, which aimed to:

- Examine the implementation, context and mechanisms of impact of the EACH-B complex intervention.
- Understand the experiences of teachers, adolescents and parents who took part in the EACH-B intervention.

## Methods and materials

### Research design

This process evaluation set out to answer three research questions, corresponding to the three core components of the MRC process evaluation guidance framework (see table 1), using a mixed-methods approach.

**Table 1:** Process evaluation research questions and corresponding data collection activities

MRC process evaluation guidance framework element	Research question	Data sources
Implementation (fidelity, dose, adaptation, reach)	To what extent were each of the EACH-B intervention components delivered as intended?	Student and teacher interviews LifeLab day observation LifeLab attendance data Smartphone application download/usage data
Mechanisms of impact	How did students, teachers and parents respond to the EACH-B intervention and what factors mediated their responses?	Student and teacher interviews Parent interviews Teacher feedback on LifeLab
Context	What organisational, social or practical factors affected the delivery, implementation and outcomes of the EACH-B intervention?	Student and teacher interviews Parent interviews School demographics

EACH-B, Engaging Adolescents in Changing Behaviour; MRC, Medical Research Council.

### Participants and procedure

The EACH-B trial recruited 49 secondary schools from the south of England, UK. The majority of schools in the trial were located in an area where rates of overweight and obesity in year 6 students were higher than the national average.^17^ Schools were randomly allocated to the intervention (n=24) or control (n=25) arm using a TENALEA randomisation procedure which was overseen by a researcher who was not involved in collection, analysis or write-up of the data. A total of 2065 students took part (intervention n=1033, control n=1032). Data were collected from students at baseline and 12-month follow-up. Full details of the study protocol are published elsewhere.^9^

A purposive sample of five intervention schools and six control schools was chosen from the EACH-B main trial to take part in the process evaluation. Schools were chosen to represent a range of different types, sizes and socioeconomic backgrounds (as detailed in table 2); however, there are some differences between the schools chosen from the intervention arm versus the control arm. This was due to an unavoidable alteration to the sampling method because of the effects of the COVID-19 pandemic. School, regional and national-level restrictions made it difficult for some schools to take part in additional research activities. Both control and intervention schools were chosen in accordance with the MRC guidance on process evaluation of complex interventions, which states that contextual factors from control schools contribute to effective evaluation. Each school took part in two ‘rounds’ of semistructured interviews; one 4–6 weeks postbaseline and one 5–6 months postbaseline. The interviews were conducted by the first author (SJ), an experienced qualitative researcher who was a PhD candidate and young woman with no prior relationship to the participants. At the start of each interview, SJ gave brief background information to participants regarding her role in the trial and the purpose of the process evaluation.

**Table 2:** School demographic characteristics

School ID	Arm of trial	Index of Multiple Deprivation (IMD) 2019 score*	% of students eligible free school meals	Attainment 8†	Academy status	Total number of students in school
1	Intervention	9	23.3	47	Non-academy	1162
2	Intervention	6	27.1	41.5	Academy	905
3	Intervention	7	37	45	Non-academy	1241
4	Intervention	9	25.6	41.4	Non-academy	624
5	Intervention	8	12.6	50.5	Non-academy	1791
6	Control	1	28.7	52.4	Non-academy	1120
7	Control	4	40.2	36.8	Academy	887
8	Control	7	17.3	45.8	Academy	1186
9	Control	5	13.6	52.1	Academy	1573
10	Control	(Unavailable‡)	22.7	(Unavailable‡)	Academy	278
11§	Control	4	12.3	49.6	Academy	1296

* 1=most deprived, 10=least deprived.

† Score out of 90 based on how well pupils have performed in up to 8 qualifications with 90 being the highest level of attainment possible.

‡ School is new and, therefore, no IMD or attainment 8 data are available.

§ school did not take part in any other process evaluation activities except parent interviews due to availability.

Students from school years 8 and 9 (aged 12–14 years) were recruited via their science teachers, who asked their class for volunteers to take part in an interview about the EACH-B study. At each ‘round’ of interviews, the research team interviewed (a) a group of students and (b) one or two (pair interview) science teachers. In ‘round 2’, heads of science (teachers who manage a school’s science department) were also interviewed where possible. The purpose of the two rounds of interviews was to compare initial and longer-term responses, to identify memorable intervention elements and explore how participant views had changed over time. Parents who had previously given their contact details were recruited via email to take part in individual online interviews. Written informed consent was obtained from all adult participants. Assent (which can be provided by children under the age of 16), combined with parental consent, was obtained for all students. Table 2 describes demographic information for each school included in the process evaluation.

#### Quantitative data

Numbers of students from each school attending the LifeLab practical day were recorded by LifeLab staff. Numbers of students who downloaded and used the app were collected throughout the trial using metadata (number of sessions per student and number of times each game element was accessed).

#### Qualitative data

Semistructured interviews were conducted online using Microsoft Teams or face-to-face in schools. For all student interviews, a teacher was also present in the room (or online) for safeguarding purposes. A total of 51 individual and group interviews with 72 students (between 5 and 15 students per school), 16 teachers (1 or 2 per school) and 11 parents (between 1 and 3 per school) were conducted, lasting an average of 22 min. Topic guides were tailored to each type of interview, with different questions for students (onlinesupplemental documents 2^5^), teachers (onlinesupplemental documents 6^9^), heads of science (onlinesupplemental documents 10  11) and parents (onlinesupplemental documents 12  13) from intervention schools and control schools.

### Impact of the COVID-19 pandemic

Following a successful pilot study in 2018–2019, the main trial began in January 2020, only to be halted when the UK entered its first national lockdown in March 2020. After a 12-month delay, the trial restarted in 2021 and data collection was completed in 2023. The main trial and process evaluation protocols were affected by continued national or local lockdowns, school closures and research team or participant absences. For example, some of the ‘round 1’ interviews with students and teachers took place soon after their baseline data collection session, but others were conducted closer to the follow-up data collection.

### Data analysis

#### Quantitative analysis

All quantitative data were compiled and summary statistics related to the research questions are presented below. These are explained alongside their presentation.

#### Qualitative analysis

All interviews were audio recorded and transcribed verbatim, then analysed using thematic analysis in NVivo. All transcripts were coded inductively, before a deductive approach was employed, where codes were refined, grouped and mapped onto the three research questions (see table 1) and the three elements of the MRC guidance framework.^16^ All transcripts were coded by at least two researchers and the coding team met weekly to discuss their interpretations of the data, ensuring that agreement was reached on each iteration of the coding frame. The wider team met monthly to discuss the analysis and ensure queries or concerns were addressed. Finally, a comprehensive coding frame was developed for each set of interviews (onlinesupplemental documents 14^17^).

## Results

### Implementation

#### To what extent were each of the EACH-B intervention components delivered as intended?

The quantitative data showed that 87.9% of eligible students visited LifeLab in person, and that an average of two teachers from each school attended professional development training (in-person or online). Professional development training was offered to all teachers from intervention schools who were directly involved in the trial (on average, two science teachers from each school).

Teachers whose students attended the LifeLab day in person also delivered planned LifeLab lessons to their students before and after their visit to complement the activities delivered at the LifeLab day, as per the study protocol. Many teachers found that these lessons contained too much content and that students were often unable to complete all activities in a lesson, which they found frustrating.

There was quite a lot of content to cover in not much time. And I know [LifeLab staff] did say, “feel free to pick and choose,” but with students in both of our classes, if there was a box to put something in, they wanted to put something in. They don’t like leaving gaps. (Teacher, school 5 (intervention), 23/03/2022)

Due to the COVID-19 pandemic, teachers from nine schools (18.4% of all schools that took part in the trial) delivered these lessons, as well as all the LifeLab practical day activities, to their students instead of attending the LifeLab facility. Flight cases (large portable containers to transport equipment to schools via courier), put together by the LifeLab team, were delivered to school and contained lesson plans, instructions, risk assessments, videos and equipment lists. These planned, packaged lessons reduced the time and resource burden on teachers, meaning they felt able to deliver the content themselves.

It was easy for me. Especially because it had been pre-planned by somebody else, and you’ve got to pick up that planning. That was very well organised, you had the lessons, you had everything. So that was… yeah, super, super useful. (Teacher, school 2 (intervention), 22/10/2021)

However, most teachers agreed that an in-person trip to LifeLab was a better opportunity for students to experience the full impact of the programme and that external visits were motivators for students to engage with science.

We couldn’t really do it the justice that it has when it’s at LifeLab…it’s always going to be better if they’re at LifeLab, than it would be here. (Teacher, school 4 (intervention), 27/01/2022)

Most teachers found the professional development training enjoyable and useful, but leaving the school site for training became more difficult during the pandemic due to staff absences and lack of support.

I think it would probably be okay, in terms of us being “allowed” to go…but budgets are tight, and cover’s tight. I’ve had several emails saying ‘we’ve got no teachers, and there’s no supply staff available, all the agencies are booked out.’ So, you know, they’re a little bit reluctant from that point of view. (Teacher, school 9 (control), 22/06/2022)

Similarly, many teachers struggled to implement the communication skills they had learnt in the training when back in school due to lack of time and opportunity. Most of their conversations with students lasted 5 min or less and happened in small groups rather than one-to-one. Teachers who organised extracurricular activities with students found it easier to initiate conversations.

We run a Duke of Edinburgh Award programme here and so when we’re on day walks and training with students, we actually do have time to have some quite different conversations with student about a wide range of things. (Teacher, school 4 (intervention), 27/01/2022)

### Mechanisms of impact

#### How did students, teachers and parents respond to the EACH-B intervention and what factors mediated their responses?

The app download data showed that 473 of 1033 eligible students (45.8% of students in the intervention arm) downloaded the app, and 228 students (48.2% of students who downloaded the app) engaged with one of the app games/activities at least once, for an average of 17.7 min per session across 2.2 sessions.

Most students said, when interviewed, that they had not used the app regularly since they first downloaded it because it was not as appealing as other games and activities, both digital and non-digital. Students suggested that social or competitive elements could make the app more enticing and gave examples of existing apps with leaderboard or messaging features.

It’s not the best game, like there are a lot better things you could do. Like you could watch TV or go out with your friends, or you could go on a PlayStation, or whatever. (Student, school 5 (intervention), 07/07/2022)

Students who had positive responses to the app mentioned specific elements they had enjoyed and all four of the in-app games were mentioned at least once, but these students were a minority.

So the app was really cool, there were these fun games… I actually played it when I was offline as well, I did it in my spare time. (Student, school 3 (intervention), 21/03/2022)

Students particularly enjoyed LifeLab and appreciated learning about their own bodies and health. The ‘round 2’ interviews showed that they remembered specific activities they had taken part in even months after their LifeLab visit.

I thought it was quite a fun way to understand us and know us better. All the CPR, the jumping, and the height and the grip tests, like that. And also doing all the little practical activities in the booklets was quite fun. (Student, school 4 (intervention), 06/07/2022)

Students generally engaged with making personal health pledges at the end of their LifeLab visit, but most teachers did not think the students revisited their pledges after that day. By their second ‘round’ of interviews, many students could not remember their own pledges.

They all made pledges and I think they worked hard to think about what would actually affect them. But I don’t think there was a pickup afterwards of that. So, it was done, and then it was forgotten about, really (Teacher, school 1 (intervention), 11/02/2022)

Most parents who were interviewed knew very little about their child’s participation in the study despite the information that had been provided to parents by schools. This information was not given to parents in a way that resonated with them, or in a way that stood out against the vast amount of information parents are required to process about their children’s lives.

[Interviewer] What do you know about the EACH-B project that your child has taken part in?

Absolutely nothing. Would I possibly know it as something else, if she’s talked about it? Has every child talked about this? I mean, with the sample that you’ve selected, have we been selected because we should have had children attending this project?

Yes, so your child definitely has taken part. They may have mentioned LifeLab. Does that ring any bells?

No. (Parent, school 3 (intervention), 05/08/2022)

Parents discussed the ways in which they wanted to be contacted by the research team and whether a website for parents could be helpful.

Maybe if it was an external letter rather than just an electronically sent one from the school, because they do, they just give you irrelevant information constantly. (Parent, school 4 (intervention), 11/08/2022)

It would be interesting to sort of see the progress on the study as well, so I suppose any information on whatever website you can create would be really useful. (Parent, school 4 (intervention), 07/07/2022)

Parents were generally unaware that their child had been offered an app as part of the study, but most were familiar with the GENEActiv watches (accelerometers used to measure physical activity) they had seen their child wearing. Some parents felt their child had understood the importance of wearing them, whereas others felt that students would not be motivated to wear them.

It’s quite unenticing. Putting myself in his shoes, that would not be something that I would be quite keen and excited to do, because what’s in it for me? Nothing. I can’t play with it, I can’t do anything with it… It’s just it looks antiquated…rubbish and useless. (Parent, school 11 (control), 09/03/2023)

Despite their lack of awareness, most parents were keen to learn more about the study and when it was explained to them, most were enthusiastic and felt their child would have enjoyed it.

I love that. He’s very interested in science, and I believe anything that increases their knowledge about life, biology, health, anything like that, is massively important and beneficial. (Parent, school 11 (control), 09/03/2023)

### Context

#### What organisational, social or practical factors affected the delivery, implementation and outcomes of the EACH-B intervention?

When discussing food choices at home, most students said that their parents oversaw food-related decisions for the household and that they ate what their parents prepared for them. Many students said they were encouraged to help with food preparation at home, but some found food preparation to be a barrier to making healthy choices.

Like snacking, we have a load of quite unhealthy stuff, and making like beans on toast, it just takes effort. I don’t wanna be stood in the kitchen for a good like five minutes, when I could be sleeping. (Student, school 6 (control), 21/06/2021)

Students had more freedom over their food choices when out with friends, but in those moments they often chose less healthy food that was cheaper and more convenient.

When I’m out with my friends my diet is not good. I normally go for McDonald’s… it’s just cheap, isn’t it? It’s just there, and it’s close to you, and there’s like one pretty much everywhere you go. (Student, school 7 (control), 12/01/2022)

Furthermore, many students felt that the environments they were exposed to by adults around them made making healthy choices difficult.

At church at youth group we have like KFC or pizza like every week. So I try not to eat like that every time I go, but I usually do. (Student, school 6 (control), 21/06/2021)

Students and teachers expressed a desire for more affordable healthy options in schools. Teachers felt that food available in school canteens did not align with what the students were being taught about the importance of a healthy diet.

From my point of view there is no link between what they hear about in the classroom and what’s actually happening downstairs in the canteen. (Teacher, school 10 (control), 13/07/2023)

Students were aware that healthy eating and physical activity were encouraged in school but struggled to name specific initiatives or campaigns. Teachers and students felt that physical activity, in the form of PE (Physical Education) lessons, extracurricular clubs, sports days and activities like inter-house competitions, was encouraged more than healthy eating.

They do encourage us to do more activity, so we were offered like cadets, clubs, all this other sort of stuff. But then I, I do find it a bit counterproductive that some of the stuff in the canteen, if you had like more than one or two a day, I feel like you would have a heart attack. (Student, school 8 (control), 24/03/2023)

The experiences of the students who took part in EACH-B were likely impacted by the investment their teachers had in the research. Though all teachers who took part were enthusiastic and spoke highly of the project, individual school environments and cultures meant that teachers in different schools experienced a range of stressors including staff shortages, curriculum pressures, post-COVID-19 behavioural and social issues and high levels of staff and student absences. Some schools were financially and practically better placed to deal with these challenges than others, and teachers who were able to prepare their colleagues and students to take part in the research felt that this helped the students engage with the activities.

I’d recorded a PowerPoint assembly to send out to tutors already… so they [knew] that this was coming… so that really helped them to engage with it as well, ‘cause they knew that it wasn’t a normal lesson. (Teacher, school 1 (intervention), 21/07/2021)

## Discussion

### Summary of findings

This process evaluation aimed to explore the delivery and implementation of the multicomponent EACH-B intervention using qualitative and quantitative methods. The in-person trip to the LifeLab facility was the highlight of the intervention for many students and teachers. The experiential learning provided by LifeLab improves health literacy, and in turn health behaviours, by encouraging young people to understand and reflect on why health is important and how it can be improved.^18 19^ It is a neutral environment where students can benefit from enjoyable, out-of-school learning that is consistent with both the science National Curriculum and the Gatsby Benchmarks.^20^ Travelling out of school to an external venue provided a unique opportunity for students to engage with the EACH-B intervention in a fun and meaningful way, to experience how science is done and to learn about health in a new and exciting environment. Out-of-school learning has been shown to build on content taught inside school and introduce students to the world of scientific research in an authentic context.^21^ It has also been associated with better social well-being,^22^ higher levels of intrinsic motivation^23^ and higher levels of physical activity.^24^ Benefits of LifeLab to teachers included the professional development training and ongoing support from the LifeLab staff to deliver the pre-LifeLab and post-LifeLab lessons (or the full LifeLab day via flight cases during the pandemic).

The remote version of LifeLab that the nine schools who took part during the pandemic experienced was likely not as effective or enjoyable as the full out-of-school LifeLab experience. This was one example of the disruption caused by the COVID-19 pandemic, which was the most significant challenge to the delivery of the EACH-B intervention. The research team was forced to adopt a pragmatic approach in response to regional, national and school-level COVID-19 restrictions, which also meant that several teachers attended the professional development training online. Review evidence suggests that the most effective interventions are delivered by teachers who receive CPD (Continuing Professional Development) training face-to-face,^8^ so the remote delivery of the LifeLab professional development day may well have indirectly impacted students’ experiences of the intervention. Increased absences and students experiencing mental health issues, and lack of staff retention in schools meant that for many teachers, engaging with research fell below more immediate priorities. Given the references from students and teachers in this study to a lack of support at school level—for example, lack of healthy food options in school canteens—it may be that improvements to school policies could provide teachers and students with much needed support to make healthier choices. Teachers were focused on supporting their most vulnerable students and enforcing COVID-19 restrictions, which led to delays when organising data collection or intervention activities with some schools. There were, however, only four schools that dropped out of the trial between the recruitment and baseline phases, which is testament to the dedication and determination of the teachers and the research team.

Students felt that the app lacked opportunities to monitor their progression or compete with peers, and many found the design and format too childish. Despite being developed in collaboration with young people, most students did not engage with the app, in part because they were not supported or motivated to revisit the app after downloading it. It is important to acknowledge that the use of a person-based approach and codesign in intervention development does not necessarily guarantee that an intervention will be successful.^25^ One approach that was not tested in the initial codesign phases of the intervention development was the use of compulsion loop theory^26^ in the app design process, which involves embedding triggers and rewards to motivate users to engage with an app consistently. App development experts can support researchers to incorporate such features to enable participants to repeatedly engage and obtain the greatest possible benefit from app-based interventions.

Most parents were keen to learn more about the research and why their child had been asked to take part and felt they had not received sufficient information from the research team. Some parents found the volume of information they received from their child’s school overwhelming and reported that alternative formats, such as physical letters instead of emails, may be more likely to capture their attention. Given the lack of parent knowledge of the app, it is possible that involving parents in their child’s use of the app may have reinforced the value of the app to students, but this may also have deterred students from engaging with it.

### Strengths and limitations

A key strength of this process evaluation was the close adherence to the MRC guidance on process evaluation of complex interventions.^16^ The guidance framework informed the study design, data collection, analysis and write-up phases and ensured that a rigorous evaluation was conducted. Another strength was the longitudinal approach to compare participant responses over time, which provided insights into participants’ longer-term engagement with the intervention. Another strength was the qualitative dataset (N=99), which was relatively large compared with previously published qualitative process evaluation studies^27 28^ and which captured participants from a range of socioeconomic, ethnic and academic backgrounds.

The main limitation was the impact of the COVID-19 pandemic, which meant that modes of data collection varied throughout this process evaluation. Some interviews were conducted online and in these cases, some students found it difficult to engage with the interview questions. Another limitation is the lack of observational findings. The initial process evaluation plan included observations of teachers in school using the skills they had learnt in the professional development training. However, due to the pandemic as well as logistical issues around school timetabling and teacher workloads, the research team was unable to conduct these observations.

### Implications and future work

Schools are ideal environments to deliver health interventions and teachers play a crucial role in supporting students to engage with behaviour change.^8^ However, high workloads combined with lack of time, resources and training in schools mean that teachers must be adequately supported by researchers to facilitate intervention delivery^29^ and that interventions must fit in with schools’ current priorities. Since the COVID-19 pandemic, teachers have also been faced with providing additional support to students with mental health issues,^30^ and students who have struggled to catch up following long periods of at-home learning.^31^ Researchers can support schools through monetary incentives, such as the £1000 each school was paid for taking part in EACH-B, and teachers should be provided with ongoing support in the form of training and resources.

It is also important that students benefit from their participation in such interventions. Young people who have taken part in research describe benefits of feeling that they are making a positive difference to others, feeling part of a safe and comfortable research environment and being able to earn money in exchange for their participation.^32^ Though individual students were not paid to take part in EACH-B, students expressed the genuine sense of satisfaction they received from knowing they were contributing to important research to help young people in the future.

The more challenging aspect of students’ participation in EACH-B was encouraging them to engage with the app. This study demonstrates the fundamental difficulty in motivating a population such as young people, who tend overall to be healthy, to engage with health improvement tools. Existing health apps that appear to be most effective are those designed to support young people with particular health conditions such as cancer^33^ and type 1 diabetes.^34^ Without the motivation of having to manage a health condition, students will likely require an alternative compulsion loop to engage with an app. A compulsion loop is a series of events and actions that encourage repeated engagement with an app or game.^26^ This loop usually results from a trigger (eg, a prompt from teachers for students to spend 10 min using their app during a science lesson), which then leads to an action (eg, the students open the app and complete one level of the ‘Gutsy’ game), which leads to a reward (eg, the students receive points for completing a level of the game), which leads to investment (eg, the students see their scores on a leaderboard within the app, which allows them to compete with their classmates), which triggers anticipation for the next reward (eg, more points), which causes the cycle to repeat.^26^ These simple yet effective design elements could be reinforced by a stronger focus on teacher and parent involvement in encouraging students to engage with app-based intervention elements. Importantly, however, compulsion loops are designed to increase app usage, but do not necessarily improve engagement with behaviour change. Further work is necessary to determine how students can be supported to use apps for meaningful, sustained health behaviour change that aligns with their own personal goals and values,^35^ as well as helping young people navigate or instigate changes to the unhealthy food environments to which they are repeatedly exposed,^36^ and which were described by participants of this study as posing additional challenges to young people’s health and well-being.

There is also a practical issue which limits the appeal of smartphone apps to young people that are designed to deliver research interventions. Academics are unlikely to be able to match the pace at which digital industries develop new software and keep up with contemporary trends because they do not have the resources to continually update apps or respond to engagement metrics and user feedback in real time.^37^ However, unlike industry developers, researchers do have the knowledge, opportunity and inclination to develop apps with health behaviour change in mind and have access to large populations of research participants. The most effective app-based interventions will take advantage of collaborations between academics and industry developers.

Parental involvement in interventions that aim to improve adolescent health behaviours should be carefully considered. Review evidence suggests that parental involvement in adolescent health interventions plays a significant role in increasing the efficacy of interventions.^13^ Evidence from school trials, however, suggests that engagement and involvement of parents is ‘a highly challenging component of health promoting schools interventions’.^38^ This is evidenced by the parent interviews, in which many parents were not aware of what their child had done as part of their participation in the EACH-B trial. Parents generally receive large amounts of communication from schools which can be overwhelming, so it is unsurprising that some of the EACH-B related information was not attended to. Future trials should work with parents from the outset to identify the most appropriate and helpful ways to keep them informed of trial progress and consider ways of facilitating parental support of young people in making health behaviour changes.

## Conclusions

Despite the challenges of working with schools, they remain valuable settings for health interventions to reach large, diverse groups of adolescents who are primed for learning. The school environment allows researchers and teachers to deliver interventions collaboratively, combining researchers’ expertise in engaging adolescents in research with teachers’ pedagogical understanding. Combining in-school intervention delivery with out-of-school experiential learning, such as LifeLab, helps teachers and students understand the importance of collaborative approaches in health improvement interventions. Collaboration is also important for app design: researchers should seek support from academic and industry partners to maximise participant engagement with apps. Prompts from parents or teachers and compulsion loops embedded into apps may provide additional motivation, but ultimately, engaging adolescents in changing their health behaviours requires support from schools, teachers, parents, researchers and technology, coming together to support adolescents to achieve goals that are important to them.

## Supplementary material

10.1136/bmjph-2024-002491 online supplemental file 1

10.1136/bmjph-2024-002491 online supplemental file 2

10.1136/bmjph-2024-002491 online supplemental file 3

10.1136/bmjph-2024-002491 online supplemental file 4

10.1136/bmjph-2024-002491 online supplemental file 5

10.1136/bmjph-2024-002491 online supplemental file 6

10.1136/bmjph-2024-002491 online supplemental file 7

10.1136/bmjph-2024-002491 online supplemental file 8

10.1136/bmjph-2024-002491 online supplemental file 9

10.1136/bmjph-2024-002491 online supplemental file 10

10.1136/bmjph-2024-002491 online supplemental file 11

10.1136/bmjph-2024-002491 online supplemental file 12

10.1136/bmjph-2024-002491 online supplemental file 13

10.1136/bmjph-2024-002491 online supplemental file 14

10.1136/bmjph-2024-002491 online supplemental file 15

10.1136/bmjph-2024-002491 online supplemental file 16

10.1136/bmjph-2024-002491 online supplemental file 17

## Data Availability

Data are available on reasonable request.
