# Supplementary material for: Mixed-methods process evaluation of the EACH-B intervention in UK secondary schools: Delivery fidelity, stakeholder responses and contextual influences
Source: BMJ Public Health. 2025 Oct 21;3(2):e002491. doi: 10.1136/bmjph-2024-002491 (PMC12551551; doi:10.1136/bmjph-2024-002491)
Supplement: online supplemental file 1 [file bmjph-3-2-s001.pdf]

Supplementary material document 1: EACH-B BCTs

EACH-B Behaviour change techniques (BCTs)

**BCT name and number**
Description of BCT
Barriers and facilitators that it addresses (identified from literature and qualitative development work)
Example of potential app content/feature

**1.1 Goal setting (behaviour)**	
Set or agree a goal defined in terms of the behaviour to be achieved. E.g. Set a goal of daily walking (e.g. 3 miles); set a goal of eating 5 pieces of fruit per day.	
Study findings	• *Taste and texture are important to adolescents.*<br>• *Competition with other valued activities can undermine target behaviours*<br>• *Some adolescents dislike exercising alone. Others dislike overt competition.*
Example	• Allowing player to choose their own goals by offering a selection of "challenges" or "quests". (Could these be specific to their journey?)<br>• Ensuring 'quests' are easy and flexible to support choice/autonomy e.g. 'create a meal with 4 different colours' rather than prescribing set foods to eat

**1.3 Goal setting (outcome)**	
Set or agree a goal defined in terms of a positive outcome of wanted behaviour. E.g. Set a goal of building endurance/speed as an outcome of changed exercise patterns.	
Study findings	• *Long-term benefits of healthy eating not considered or valued .*<br>• *Perceived link between healthy eating and personal values (e.g. homelife, adulthood, appearance, energy) can facilitate target behaviour.*
Example	• Allowing player to choose their own overall goal when they first login, for example by selecting a specific character or journey that aligns with personal values (i.e. strong, energetic, healthy, trailblazer etc.).<br>• Allowing player to change their goal/character if they want to.

**1.4 Action planning**	
Prompt detailed planning of performance of the behaviour (must include at least one of context, frequency, duration and intensity). Context may be environmental (physical or social) or internal (physical, emotional or cognitive). E.g. Prompt planning the performance of a particular physical activity (e.g. running) at a particular time (e.g. before work) on certain days of the week.	
Study findings	• *Perceived lack of time/busy schedules – difficult to incorporate target behaviours in routine*<br>• *Competition with other valued activities can undermine target behaviours.*<br>• *Practical constraints on target behaviour: time, cost, access to facilities, weather, safety.*

Example	• Prompting player to plan how they intend to reach a goal/ complete a challenge or quest e.g. through a 'hints and tips' option.<br>• Allowing players to enlist help in their quests e.g. access a diary/calendar to plan when they can fit activities in, setting alarms or reminders, asking for help from peers/family etc.

**2.2 feedback on behaviour**	
Monitor and provide informative or evaluative feedback on performance of the behaviour (e.g. form, frequency, duration, intensity).	
Study findings	• *Can feel a lack of competence/ confidence in activity ("not very good at it")*<br>• *Achievements and streaks important, they don't want to break a streak.*<br>• *They want app and activities to be fun. They don't want to feel like they're exercising, want to do something fun, wanted rewards.*
Example	• Rewarding players for making progress and completing a quest.<br>• Providing feedback on their performance compared to their previous performance (e.g. longer streak than last time).<br>• Providing feedback on the relative effort (e.g. you have walked the length of the titanic today)<br>• Embedding behavioural support within feedback (e.g. hints and tips when progress slow; demonstrating how their progress can reinforce valued outcomes e.g. strength, being a role model to family, appearance etc.)

**2.3 self-monitoring of behaviour**	
Establish a method for the person to monitor and record their behaviour(s) as part of a behaviour change strategy. E.g. suggest that player record daily, in the app, whether they have brushed their teeth for at least two minutes before going to bed.	
Study findings	• *Some like data, tracking steps and being able to get stats about what they were doing.*<br>• *Some dislike sharing your data socially / overt competition, others dislike doing things alone.*
Example	• Allowing players to track their progress in the app: count steps, measure distance, food choices, photo diaries. (Not sure what's feasible)<br>• Allowing players the option to share data with others and compete against other players or in groups.<br>• Allowing players the option to self-monitor their own progress without sharing.

**3.2 social support (practical)**	
Advise on, arrange, or provide ***practical*** *help (e.g. from friends, relatives, teachers) for performance of the behaviour. E.g., Ask the partner of the patient to put their tablet on the breakfast tray so that the patient remembers to take it.*	
Study findings	• *Parental knowledge and habits/poor role models, but valued source of information.*<br>• *Perceived lack of control - Parents generally control home foods and often lunches.*<br>• *Availability of healthy options at home/school.*

Example	• Quests that are about getting parents to involve teen in cooking or preparing food (or getting teens to involve parents in their quests …).<br>• Quests about getting teens and parents to do something new together.<br>• Quests about getting teens to try something with their friends.<br>• Quests that friends can help complete (but not necessary to complete)

**3.3 social support (emotional)**	
Advise on, arrange, or provide emotional social support (e.g. from friends, relatives, teachers) for performance of the behaviour.	
Study findings	• *Parental habits/poor role models, but valued source of information.*<br>• *Stigmatisation of healthy food in peer group. In some groups, other groups see healthy food as a status booster.*<br>• *Social – dislike exercising alone, others dislike overt competition.*<br>• *Very important to be connected to other people. Social networking with friends, finding their friends, competing with friends.*<br>• *They don't want to feel like they're alone, want support. Having an anonymous support network.*
Example	• Quests that are about getting parents to allow teen to cooking or preparing food.<br>• Allowing player to connect with friends in the app in a way that does not involve competition.<br>• Allowing player the option to encourage/support others (peers and family) and to receive encouragement/support from others (peers and family). (likes, thumbs up, gold stars etc)

**4.1 instruction on how to perform the behaviour**	
Advise on how to perform the behaviour. E.g. advise how to chop a carrot into sticks; advise how to make cheap & healthy snacks.	
Study findings	• *Perceived higher cost of healthy options. Desire value for money and "more filling" foods like pizza and Paninis seen as better choice for that.*<br>• *Taste and texture are important to adolescents.*<br>• *PA is seen as effortful.*<br>• *Some adolescents feel a lack of competence/confidence in doing activity.*<br>• *Wanted ideas for healthy swaps, the app to suggest things to eat and tell them how well or not well they've been eating that day.*
Example	• Offering a clickable link to "more information" or "tips" on how to perform the task or ideas for how it can be achieved.<br>• Allowing players to submit their own solutions to how to complete tasks and make them easier.

**4.4 behavioural experiments**
Advise on how to identify and test hypotheses about the behaviour, its causes and consequences, by collecting and interpreting data.

Study findings	• *Perceived higher cost of healthy options.*<br>• *Taste and texture are important to adolescents.*<br>• *PA is seen as effortful.*<br>• *Some adolescents feel a lack of competence/confidence in doing activity.*<br>• *Reluctance to make long-term commitment.*
Example	• Offering "quests" that are about "myth busting": encouraging experimentation related to main barriers such as taste, texture, cost of foods etc.<br>• Offering "quests" that are framed as "experiments" for them to test things out and record their observations.<br>• Encouraging players to reflect on the challenge they completed, e.g. how did they feel, how challenging was it, how accomplished do they feel as a result, how likely is it they might try something similar in the future? (N.B. easy to complete reflections e.g. rating scales, rather than forced diary-style entry)

**5.1 Information about health consequences**	
Provide information (e.g. written, verbal, visual) about health consequences of performing the behaviour.	
Study findings	• *Stigmatisation of healthy food in peer group. In some groups, other groups see healthy food as a status booster.*<br>• *Long-term benefits of healthy eating not considered or valued.*
Example	• Offering a clickable link to "more information" on the health value of being active and eating fruits and vegetables (not dieting) (e.g. what's the science?).<br>• Links to youtube videos discussing the health implications of eating well and being active when you're a teen.<br>• Health information content ideally mostly framed to more immediate benefits, e.g. acne reduction, good skin, good hair, good nails, improved cognitive performance, better sleep, more energy.<br>• Embed information in feedback on performance

**5.3 Information about social and environmental consequences**	
Provide information (e.g. written, verbal, visual) about social and environmental consequences of performing the behaviour. E.g. Inform a smoker that the majority of people disapprove of smoking in public places.	
Study findings	• *Parental habits/poor role models, but valued source of information*<br>• *Stigmatisation of healthy food in peer group. In some groups, other groups see healthy food as a status booster.*<br>• *Long-term benefits of healthy eating not considered or valued.*<br>• *Competition with other valued activities*
Example	• Offering a clickable link to "more information" on the social and environmental value of being active and eating fruits and vegetables (not dieting).<br>• Links to youtube videos discussing the social and environmental value of health behaviours.<br>• Allow users to endorse challenges they enjoyed by "liking" them.

**5.4 monitoring of emotional consequences**	
Prompt assessment of feelings after attempts at performing the behaviour. E.g. prompt person to record how they feel after taking their daily walk.	
Study findings	• *Sedentary routines*<br>• *Tiredness/fatigue*<br>• *Feeling a lack of competence/ confidence in activity*
Example	• Encouraging players to reflect on the challenge they completed, e.g. how did they feel, how challenging was it, how accomplished do they feel as a result, how likely is it they might try something similar in the future?

**6.1 Demonstration of the behaviour**	
Provide an observable sample of the performance of the behaviour (e.g. via film, pictures, for the person to aspire to or imitate), includes 'Modelling'.	
Study findings	• *Perceived higher cost of healthy options.*<br>• *Practical constraints: time, cost, access to facilities, weather, safety.*<br>• *Ignoring healthy options at corner shops where YP mostly buy snacks.*
Example	• Offering a clickable link to "tips" videos on how to perform the task or ideas for how it can be achieved. E.g., offering clickable link to YouTube videos about how to prep healthy snacks for school.<br>• Allowing players to save and/or share YouTube videos to help complete tasks and make them easier.

**6.2. Social comparison (optional)**	
Draw attention to others' performance to allow comparison with the person's own performance. Note: being in a group setting does not necessarily mean that social comparison is actually taking place.	
Study findings	• *Some adolescents like competitive elements.*<br>• *Involvement of social influencers – parents, teachers, peers*<br>• *Very important to be connected to other people. Social networking with friends, finding their friends, competing with friends.*
Example	• Offering the option to follow others and compete with others on challenges.<br>• Offering the option to compete as a group.<br>• Offering the option to share progress and view other's progress if they have allowed this.

**6.3. Information about others' approval**
Provide information about what other people think about the behaviour. The information clarifies whether others will like, approve or disapprove of what the person is doing or will do.

Study findings	• *Involvement of social influencers – parents, teachers, peers*<br>• *Some dislike sharing your data socially, others dislike doing things alone*<br>• *Very important to be connected to other people. Social networking with friends, finding their friends, competing with friends.*
Example	• Allowing player to connect with friends in the app in a way that does not involve competition.<br>• Offering information about how many other people have selected a challenge.<br>• Offering ability to endorse/like challenges.<br>• Allowing player the option to encourage/support others and to receive encouragement/support from others. (likes, thumbs up, gold stars etc)

**8.1 Behavioural practice and rehearsal<br>8.2 behaviour substitution**	
8.1 Prompt practice or rehearsal of the performance of the behaviour one or more times in a context or at a time when the performance may not be necessary, in order to increase habit and skill.<br>8.2 Prompt substitution of the unwanted behaviour with a wanted or neutral behaviour.	
Study findings	• *Perceived higher cost of healthy options.*<br>• *Feeling a lack of competence/ confidence in activity ("not very good at it")*<br>• *Sedentary routines*
Example	• Themed challenges: series of challenges about prepping lunch or snacks, but with varying themes/ seasons etc (e.g. challenges for home, school, weekends, on-the-go etc.).<br>• Themed challenges: series of challenges about food swap experiments.

**8.7. Graded tasks**	
Set easy-to-perform tasks, making them increasingly difficult, but achievable, until behaviour is performed.	
Study findings	• *Reluctance to make long-term commitment*<br>• *Feeling a lack of competence/ confidence in activity ("not very good at it")*<br>• *Feelings of shame / embarrassment (induced by intervention)*
Example	• Themed challenges: series of challenges that build up abilities little by little.<br>• Challenges that get "harder" as you "level up" /progress in your journey.

**10.9. Self-reward**	
*Prompt self-praise or self-reward if and only if there **has been** effort and/or progress in performing the behaviour. E.g. Encourage to reward self with material (e.g., new clothes) or other valued objects if and only if they have adhered to a healthy diet.*	
Study findings	• *Motivation – competition with other valued activities*<br>• *Motivation – PA is effortful*<br>• *Sedentary routines*<br>• *Tiredness/fatigue*<br>• *Adolescents wanted rewards.*

Example	• Prompt adolescents to reward themselves when they have done well.<br>• Offer clickable link to "suggestions" for rewards, or allow others to suggest rewards.

**12.1 restructuring the physical environment**	
*Change, or advise to change the **physical** environment in order to facilitate performance of the wanted behaviour or create barriers to the unwanted behaviour.*	
Study findings	• *Availability of healthy options at home/school*<br>• *Availability of healthy options at corner shops where YP mostly buy snacks*<br>• *Practical constraints: time, cost, access to facilities, weather, safety*<br>• *Tiredness/fatigue*
Example	• Challenges that focus on exploring the immediate environment, e.g. Find the places in the shop where they put the snacks so you can most easily see them.<br>• Challenges about identifying the "risks" and "triggers" in your environment e.g. in the kitchen.

**12.2 restructuring the social environment**	
*Change, or advise to change the **social** environment in order to facilitate performance of the wanted behaviour or create barriers to the unwanted behaviour.*	
Study findings	• *Parental habits/poor role models, but valued source of information*<br>• *Stigmatisation of healthy food in peer group. In some groups, other groups see healthy food as a status booster.*<br>• *Perceived lack of control - Parents generally control home foods and often lunches*<br>• *Motivation – competition with other valued activities*
Example	• Challenges that focus on the social dynamic between player and their friends and family. Rejigging power dynamics.<br>• Challenging parents to do something silly or to give over control of the kitchen for one night.

**13.1 Identification of self as role model**	
Prompt the person to view self as someone who may be an example to others.	
Study findings	• *Parental habits/poor role models, but valued source of information.*<br>• *Stigmatisation of healthy food in peer group. In some groups, other groups see healthy food as a status booster.*<br>• *Newfound power and freedom of adolescence.*

Example	
Example	• Framing the game characters as role models that others look up to and aspire to be like.<br>• Allowing the other players to follow you or like your achievements (optional).<br>• Position the players as role models who are showing others the way, making a difference etc through the language and phrasing used in text-aspects of the game.

**13.4. Valued self-identify**	
Write or complete rating scales about a cherished value or personal strength as a means of affirming the person's identity as part of a behaviour change strategy	
Study findings	• *Adolescents feel existing apps are not relevant to them personally.*<br>• *Value of healthy food depends on peer group.*<br>• *Long-term benefits of healthy eating not considered or valued.*<br>• *Perceived link between healthy eating and personal values (e.g. homelife, adulthood, appearance, energy).*
Example	• Allowing player to choose a character and/or journey to be chosen at the start that represents their core values and personal strengths, and identifies a “health journey” relevant to them.<br>• Tailored messaging/ rewards/ challenges to their “preferences”

**13.2 framing/reframing**	
*Suggest the deliberate adoption of a perspective or new perspective on behaviour (e.g. its purpose) in order to change cognitions or emotions about performing the behaviour (includes '**Cognitive structuring**'). E.g. Suggest that the person might think of the tasks as reducing sedentary behaviour (rather than increasing activity).*	
Study findings	• *Perceived higher cost of healthy options. Desire value for money and “more filling” foods like pizza and paninis seen as better choice for that.*<br>• *Stigmatisation of healthy food in peer group. In some groups, other groups see healthy food as a status booster.*<br>• *They want app and activities to be fun. They don’t want to feel like they’re exercising, want to do something fun.*
Example	• Variation how the challenges are framed i.e. what they are about.<br>• Themed challenges: series of challenges about prepping lunch or snacks, but with varying themes/ seasons etc.<br>• Themed challenges: series of challenges about food swap experiments.

**15.1 verbal persuasion about capability**
Tell the person that they can successfully perform the wanted behaviour, arguing against self-doubts and asserting that they can and will succeed.

Study findings	• *Feeling a lack of competence/ confidence in activity (“not very good at it”).*<br>• *Motivation – PA is effortful.*<br>• *Perceived lack of time/busy schedules – difficult to incorporate in routine.*
Example	• Throwback to a successful challenge or best streak in the past.<br>• Direct encouraging messaging.<br>• Ability to encourage other players through stickers or messages.

**14.4. Reward approximation**	
Reward following any approximation to the target behaviour, gradually rewarding only performance closer to the wanted behaviour.	
Study findings	• *Feeling a lack of competence/ confidence in activity (“not very good at it”).*<br>• *Motivation – PA is effortful.*<br>• *Perceived lack of time/busy schedules – difficult to incorporate in routine.*
Example	• Acknowledge progress towards goal and reward partial “points”<br>• Forfeit task: offer choice to perform a forfeit task to earn bonus points

**15.3 Focus on past success**	
Prompt to think about or list previous successes in performing the behaviour (or parts of it).	
Study findings	• *Feeling a lack of competence/ confidence in activity (“not very good at it”).*<br>• *Motivation – PA is effortful.*<br>• *Perceived lack of time/busy schedules – difficult to incorporate in routine.*
Example	• Prompt to think about previous success at behaviour in a challenge.<br>• Throwback to a successful challenge or best streak in the past.

**15.4. Self-talk**	
Prompt positive self-talk (aloud or silently) before and during the behaviour.	
Study findings	• *Feeling a lack of competence/ confidence in activity (“not very good at it”).*<br>• *Motivation – PA is effortful.*<br>• *Perceived lack of time/busy schedules – difficult to incorporate in routine.*
Example	• Challenges about mantras and mindfulness that involve positive self-talk.
