# Supplementary material for: Mixed-methods process evaluation of the EACH-B intervention in UK secondary schools: Delivery fidelity, stakeholder responses and contextual influences
Source: BMJ Public Health. 2025 Oct 21;3(2):e002491. doi: 10.1136/bmjph-2024-002491 (PMC12551551; doi:10.1136/bmjph-2024-002491)
Supplement: online supplemental file 2 [file bmjph-3-2-s002.pdf]

# Supplementary material document 2: Student topic guide round 1 control schools

**EACH-B process evaluation interviews: Semi-structured topic guide**

**INTRODUCTION**

Hello, I'm *[insert name]* from the University of Southampton & I'll be interviewing you today. Before we get started, I'd just like to run through a few things with you. We want to know how people who have taken part in EACH-B have found the experience, and if you think there is anything we could change or improve on. I'm going to be asking you about how you have found the study and what you think about being involved in research. Our chat won't last for more than 20 or 30 minutes and you are free to leave at any time. We would like to audio-record this interview, and this will be typed up, read only by us in the research team and your name will be taken off the written version.

**Consented to audio recording:** **Yes / No** (circle)

[Ensure that the participant is happy to continue and has provided consent – ensure it is **INITIALED**]

## EACH-B

- What did you understand about why we are doing the study and what it's all about?
- What did you think about being part of EACH-B when you first heard about it?

## Baseline

- How did you find filling in the questionnaires?
- What do you remember most about filling in the questionnaires?
- What did you understand about why we asked you to fill in those questionnaires?
- What do you think could have been done differently?

- How did you find wearing the Geneactiv?
- How often did you take it off?
- What were the most common reasons for taking it off?

## Life in General

- What do you generally like to eat?
- What do you eat at home?
- What do you eat outside of home?
- How active are you generally?
- Have there been any changes to what you eat or how active you are in the last few months?
- Have there been any changes at school in the last few months that might relate to your health and wellbeing?

**Many thanks for your time.**
