# Supplementary material for: Mixed-methods process evaluation of the EACH-B intervention in UK secondary schools: Delivery fidelity, stakeholder responses and contextual influences
Source: BMJ Public Health. 2025 Oct 21;3(2):e002491. doi: 10.1136/bmjph-2024-002491 (PMC12551551; doi:10.1136/bmjph-2024-002491)
Supplement: online supplemental file 3 [file bmjph-3-2-s003.pdf]

Supplementary material document 3: Student topic guide round 1 intervention schools

EACH-B process evaluation interviews: Semi-structured topic guide

INTRODUCTION

Hello, I'm *[insert name]* from the University of Southampton & I'll be interviewing you today. Before we get started, I'd just like to run through a few things with you. We want to know how people who have taken part in EACH-B have found the experience, and if you think there is anything we could change or improve on. I'm going to be asking you about how you have found the study and what you think about being involved in research. Our chat won't last for more than 20 or 30 minutes and you are free to leave at any time. We would like to audio-record this interview, and this will be typed up, read only by us in the research team and your name will be taken off the written version.

Consented to audio recording: **Yes / No** (circle)

[Ensure that the participant is happy to continue and has provided assent, and parental consent – ensure it is **INITIALED**]

Baseline (~10 minutes)

- How did you find filling in the questionnaires?
- What do you remember most about filling in the questionnaires?
- What did you understand about why we were asking you to fill in those questionnaires?
- What do you think could have been done differently?
- How did you find wearing the activity tracker?
- How often did you take it off?
- What were the most common reasons for taking it off?

App (~5 minutes)

- Who has downloaded the app? (show of hands)
- ***If yes…***
- How easy or difficult was it to download the app?
- How have you found the app?
- How much have you been using the app?
- What do you like the most/least about the app?
- How do you think the app has helped you to eat more healthily or exercise more?
- What could be improved about the app?

- ***If no…***
- What has stopped you from downloading the app?
- What would help you to download the app/change your mind?

Flight cases and lifelab lessons/trip

- What did you think of the LifeLab lessons/LifeLab trip (delete as appropriate)?
- What was the best/worst thing about the lessons/trip?
- What do you think was the main aim of EACH-B?

Parents (~5 minutes)

- How much have your parents been involved in you taking part in the study?
- Are you aware that there is a website for parents?
- Have your parents used this website?

Many thanks for your time.
