# Supplementary material for: Mixed-methods process evaluation of the EACH-B intervention in UK secondary schools: Delivery fidelity, stakeholder responses and contextual influences
Source: BMJ Public Health. 2025 Oct 21;3(2):e002491. doi: 10.1136/bmjph-2024-002491 (PMC12551551; doi:10.1136/bmjph-2024-002491)
Supplement: online supplemental file 4 [file bmjph-3-2-s004.pdf]

Supplementary material document 4: Student topic guide round 2 control schools

EACH-B process evaluation interviews: Semi-structured topic guide

INTRODUCTION

Hello, I'm *[insert name]* from the University of Southampton & I'll be interviewing you today. Before we get started, I'd just like to run through a few things with you. We want to know how people who have taken part in EACH-B have found the experience, and if you think there is anything we could change or improve on. I'm going to be asking you about how you have found the study and what you think about being involved in research. Our chat won't last for more than 20 or 30 minutes and you are free to leave at any time. We would like to audio-record this interview, and this will be typed up, read only by us in the research team and your name will be taken off the written version.

Consented to audio recording: **Yes / No** (circle)

[Ensure that the participant is happy to continue and has provided consent – ensure it is **INITIALED**]

EACH-B

1. How have you found being part of the EACH-B project in general?
   Best thing? What could have made it more fun?
2. What do you remember about the baseline data collection?
3. Did you understand what a research study was before you took part in this study?
4. What are the good things about taking part in scientific research?
5. Who does a study like EACH-B benefit?
6. Would you like to take part in other studies now that you know what it's like?

Life in General

7. What do you generally like to eat?
8. How active are you generally?
9. Have there been any changes to what you eat or how active you are in the last few months?

School life

10. Have there been any changes at school in the last few months that might relate to your health and wellbeing?
    Change to food available in school? Change to PE/clubs/exercise opportunities? Mental health and wellbeing?
11. How well does school support you to stay healthy?

Many thanks for your time.
