# Supplementary material for: Mixed-methods process evaluation of the EACH-B intervention in UK secondary schools: Delivery fidelity, stakeholder responses and contextual influences
Source: BMJ Public Health. 2025 Oct 21;3(2):e002491. doi: 10.1136/bmjph-2024-002491 (PMC12551551; doi:10.1136/bmjph-2024-002491)
Supplement: online supplemental file 5 [file bmjph-3-2-s005.pdf]

Supplementary material document 5: Student topic guide round 2 intervention schools

EACH-B process evaluation interviews: Semi-structured topic guide

INTRODUCTION

Hello, I'm *[insert name]* from the University of Southampton & I'll be interviewing you today. Before we get started, I'd just like to run through a few things with you. We want to know how people who have taken part in EACH-B have found the experience, and if you think there is anything we could change or improve on. I'm going to be asking you about how you have found the study and what you think about being involved in research. Our chat won't last for more than 20 or 30 minutes and you are free to leave at any time. We would like to audio-record this interview, and this will be typed up, read only by us in the research team and your name will be taken off the written version.

Consented to audio recording: **Yes / No** (circle)

[Ensure that the participant is happy to continue and has provided assent, and parental consent – ensure it is **INITIALED**]

EACH-B

1. How have you found being part of the EACH-B project in general?
2. What have been the best bits about taking part?
3. What has changed at school since you started the EACH-B project?
4. How has being part of EACH-B changed what you think about your life and health?

LifeLab

5. What did you think of the LifeLab activities/trip?
6. What do you remember most about the LifeLab activities/trip?
7. What did you enjoy the most/least about the LifeLab activities/trip?
8. What do you remember about the pledge you made?
9. How well do you think you have kept up with the pledge?
10. What further help and support would you like to help you in making the changes you have identified in your pledge?

App

11. How often have you used the app in the last few months?
12. (if used often) What has made you keep using the app regularly?
13. (if not used often) What made you stop using the app?
14. Which bit of the app have you used the most?
15. How do you think your diet or exercise has changed since using the app?
16. What do you think we could improve about the app?

Many thanks for your time.
