# Supplementary material for: Mixed-methods process evaluation of the EACH-B intervention in UK secondary schools: Delivery fidelity, stakeholder responses and contextual influences
Source: BMJ Public Health. 2025 Oct 21;3(2):e002491. doi: 10.1136/bmjph-2024-002491 (PMC12551551; doi:10.1136/bmjph-2024-002491)
Supplement: online supplemental file 6 [file bmjph-3-2-s006.pdf]

Supplementary material document 6: Teacher topic guide round 1 control schools

EACH-B process evaluation interviews: Semi-structured topic guide

INTRODUCTION

Hello, I'm *[insert name]* from the University of Southampton & I'll be interviewing you today. Before we get started, I'd just like to run through a few things with you. We want to know how the teachers who have taken part in EACH-B have found the experience, and if you think there is anything we could change or improve on. I'm going to be asking you about how you have found the study and how you think the experience has been for your students. Our chat won't last for more than 20 minutes and you are free to leave at any time. We would like to audio-record this interview, and this will be typed up, read only by us in the research team and your name will be taken off the written version.

Consented to audio recording: **Yes / No** (circle)

[Ensure that the participant is happy to continue and has provided consent – ensure it is **INITIALED**]

EACH-B

- What is your understanding of the aim of EACH-B?
- What did your students think of EACH-B when they first heard about it?
- How did you feel about your students' reactions to the study?
- How do you feel about being involved in research like EACH-B?
- How did you feel when you found out your school was in the control arm?

Baseline

- How did you find conducting the baseline data collection?
- How well do you think the students engaged with the baseline data collection activities?
- What do you think could have been done differently?
- What, if any, issues did you have with explaining and handing out the Geneactiv devices to your students?

Consent

- How do you feel the process of getting parental consent went?
- What particular challenges were there?
- What could we do to support teachers in the consenting process?

Many thanks for your time.
