# Supplementary material for: Mixed-methods process evaluation of the EACH-B intervention in UK secondary schools: Delivery fidelity, stakeholder responses and contextual influences
Source: BMJ Public Health. 2025 Oct 21;3(2):e002491. doi: 10.1136/bmjph-2024-002491 (PMC12551551; doi:10.1136/bmjph-2024-002491)
Supplement: online supplemental file 7 [file bmjph-3-2-s007.pdf]

Supplementary material document 7: Teacher topic guide round 1 intervention schools

EACH-B process evaluation interviews: Semi-structured topic guide

INTRODUCTION

Hello, I'm *[insert name]* rom the University of Southampton & I'll be interviewing you today. Before we get started, I'd just like to run through a few things with you. We want to know how the teachers who have taken part in EACH-B have found the experience, and if you think there is anything we could change or improve on. I'm going to be asking you about how you have found the study and how you think the experience has been for your students. Our chat won't last for more than 20 minutes and you are free to leave at any time. We would like to audio-record this interview, and this will be typed up, read only by us in the research team and your name will be taken off the written version.

Consented to audio recording: **Yes / No** (circle)

[Ensure that the participant is happy to continue and has provided consent – ensure it is **INITIALED**]

<u>Baseline</u> (~8 minutes)

- How did you find the baseline data collection activities?
- How well do you think the students engaged with the baseline data collection activities?
- What do you think could have been done differently?
- What, if any, issues did you experience with explaining and handing out the Geneactiv devices to your students?

<u>Healthy Conversation Skills</u> (~8 minutes)

If attended the PD training…

- What do you remember about the Professional Development training organised by LifeLab?
- How much do you remember about the Healthy Conversation Skills training at the PD training?
- How did you find the Healthy Conversation Skills training?
- How easy or difficult has it been to use HCS in your day to day interactions with students?
- What would make it easier for you to use HCS more often with your students?

If not…

- What were the challenges in attending the PD training?
- Was the relevance of the PD training clear?
- What would help to improve PD training attendance?

<u>Consent (~8 minutes)</u>

- How do you feel the process of getting parental consent went?
- What particular challenges were there?
- What could we do to support teachers in the consenting process?

<u>Flight cases and lifelab lessons/trip:</u>

- How did you find using the flight case? Which activities did you complete/not complete?
- How easy/difficult were the instructions? What did you think of the videos to explain the practicals?
- How engaged were your students in the LifeLab lessons?
- What could we do to improve the flight case/LifeLab lessons?
- What did you think of the LifeLab trip (if applicable)? How do you think the students found the day?
- What did you/your students think of the app trailer?
- How easy/difficult was it to explain the app to your students?

Many thanks for your time.
