# Supplementary material for: Mixed-methods process evaluation of the EACH-B intervention in UK secondary schools: Delivery fidelity, stakeholder responses and contextual influences
Source: BMJ Public Health. 2025 Oct 21;3(2):e002491. doi: 10.1136/bmjph-2024-002491 (PMC12551551; doi:10.1136/bmjph-2024-002491)
Supplement: online supplemental file 8 [file bmjph-3-2-s008.pdf]

Supplementary material document 8: Teacher topic guide round 2 control schools

EACH-B process evaluation interviews: Semi-structured topic guide

INTRODUCTION

Hello, I'm *[insert name]* from the University of Southampton & I'll be interviewing you today. Before we get started, I'd just like to run through a few things with you. We want to know how the teachers who have taken part in EACH-B have found the experience, and if you think there is anything we could change or improve on. I'm going to be asking you about how you have found the study and how you think the experience has been for your students. Our chat won't last for more than 20 minutes and you are free to leave at any time. We would like to audio-record this interview, and this will be typed up, read only by us in the research team and your name will be taken off the written version.

Consented to audio recording: **Yes / No** (circle)

[Ensure that the participant is happy to continue and has provided consent – ensure it is **INITIALED**]

EACH-B

1. What did your students think of EACH-B when they first heard about it?
   Their reactions? Any reluctance?
2. Did they understand what they were going to be getting involved with?
3. How did you feel when you found out your school was in the control arm?
   Any disappointment?
4. Would you be keen to engage with the University of Southampton in future studies?
5. Would you be keen for your school to engage with LifeLab in the future?
6. What has been your main take away from taking part in this research?
7. Would you be keen to take part in other studies now that you have experienced it?

School policies

8. What, if any, policies do you have at school level that relate to health and wellbeing? (E.g. healthy school initiatives, rules around mobile phone use etc.)
9. How much does your school promote a healthy lifestyle to students?
10. How much does your school link with local groups that offer opportunities for physical activity for young people?
11. Have there been any big changes at school in the last few months that might relate to students' health and wellbeing?

Many thanks for your time.
