# Supplementary material for: Mixed-methods process evaluation of the EACH-B intervention in UK secondary schools: Delivery fidelity, stakeholder responses and contextual influences
Source: BMJ Public Health. 2025 Oct 21;3(2):e002491. doi: 10.1136/bmjph-2024-002491 (PMC12551551; doi:10.1136/bmjph-2024-002491)
Supplement: online supplemental file 9 [file bmjph-3-2-s009.pdf]

Supplementary material document 9: Teacher topic guide round 2 intervention schools

EACH-B process evaluation interviews: Semi-structured topic guide

INTRODUCTION

Hello, I'm *[insert name]* from the University of Southampton & I'll be interviewing you today. Before we get started, I'd just like to run through a few things with you. We want to know how the teachers who have taken part in EACH-B have found the experience, and if you think there is anything we could change or improve on. I'm going to be asking you about how you have found the study and how you think the experience has been for your students. Our chat won't last for more than 20 minutes and you are free to leave at any time. We would like to audio-record this interview, and this will be typed up, read only by us in the research team and your name will be taken off the written version.

Consented to audio recording: **Yes / No** (circle)

[Ensure that the participant is happy to continue and has provided consent – ensure it is **INITIALED**]

LifeLab

1. What do you remember about teaching the LL activities/pre-lessons?
2. How easy or difficult did you find it to deliver the LL activities using the flight case/the pre lessons?
3. What did your students enjoy the most/least about the LifeLab lessons/LifeLab trip?
4. How meaningful do you think the pledges were that they made?
5. What elements of the Lifelab curriculum have you been able to implement since you delivered the lessons? What would make this easier?
6. What do you think have been the key benefits from participation in the LifeLab programme for you and your students?

Healthy Conversation Skills

7. What do you remember about the Professional Development training organised by LifeLab?
8. How much do you remember about the Healthy Conversation Skills training? How did you find it?
9. How easy or difficult has it been to use HCS in your day to day interactions with students? Do you think HCS is relevant to your role?
10. What would make it easier for you to use HCS more often with your students?

Student engagement with EACH-B

11. How well have the students have engaged with the app since downloading it?
12. What is your sense of how the students found the app?
13. What is your sense of whether the app has influenced their diet or physical activity?
14. Do you think the app has value as part of this intervention?
15. What would make it easier for you to work with us on a trial like this?
16. What could we have done differently to make it easier for you as a school to take part?

Many thanks for your time.
