# Supplementary material for: Mixed-methods process evaluation of the EACH-B intervention in UK secondary schools: Delivery fidelity, stakeholder responses and contextual influences
Source: BMJ Public Health. 2025 Oct 21;3(2):e002491. doi: 10.1136/bmjph-2024-002491 (PMC12551551; doi:10.1136/bmjph-2024-002491)
Supplement: online supplemental file 11 [file bmjph-3-2-s011.pdf]

Supplementary material document 11: Heads of science topic guide round 2 interention schools

EACH-B process evaluation interviews: Semi-structured topic guide

INTRODUCTION

Hello, I'm *[insert name]* from the University of Southampton & I'll be interviewing you today. Before we get started, I'd just like to run through a few things with you. We want to know how the teachers who have taken part in EACH-B have found the experience, and if you think there is anything we could change or improve on. I'm going to be asking you about how you have found the study and how you think the experience has been for your students. Our chat won't last for more than 20 minutes and you are free to leave at any time. We would like to audio-record this interview, and this will be typed up, read only by us in the research team and your name will be taken off the written version.

Consented to audio recording: **Yes / No** (circle)

[Ensure that the participant is happy to continue and has provided consent – ensure it is **INITIALED**]

EACH-B

1. How do you feel your teachers have found being part of EACH-B?
2. How well do you think the students have engaged with the project?
3. How much do you think students' daily experience of school has been influenced by the school taking part in EACH-B?
4. What would make it easier for you to work with us on a trial like this?
5. What could we have done differently to make it easier for you as a school to take part?

Healthy Conversation Skills

6. How much do you know about the training teachers received as part of EACH-B?
7. What aspects of this training have teachers implemented in their daily work?
8. How much do you know about Healthy Conversation Skills, the communication skills training provided as part of EACH-B?

School policies

9. What, if any, policies do you have at school level that relate to health and wellbeing? (E.g. healthy school initiatives, rules around mobile phone use etc.)
10. How much does your school promote a healthy lifestyle to students?
11. How much does your school link with local groups that offer opportunities for physical activity for young people?
12. Have there been any major changes at your school since EACH-B started?

Staff movement between schools

13. Have you had any new science teachers join your school since you conducted the baseline data collection?
14. Have you had any science teachers leave your school since you conducted the baseline data collection?

Many thanks for your time.
