# Supplementary material for: Mixed-methods process evaluation of the EACH-B intervention in UK secondary schools: Delivery fidelity, stakeholder responses and contextual influences
Source: BMJ Public Health. 2025 Oct 21;3(2):e002491. doi: 10.1136/bmjph-2024-002491 (PMC12551551; doi:10.1136/bmjph-2024-002491)
Supplement: online supplemental file 12 [file bmjph-3-2-s012.pdf]

Supplementary material document 12: Parent topic guide control schools

EACH-B process evaluation interviews: Semi-structured topic guide

INTRODUCTION

Hello, I'm *[insert name]* from the University of Southampton & I'll be interviewing you today. Before we get started, I'd just like to run through a few things with you. We want to know how the parents of children who have taken part in EACH-B have found the experience, and if you think there is anything we could change or improve on. I'm going to be asking you about how you have found the study and how you think the experience has been for your children. Our chat won't last for more than 20 minutes and you are free to leave at any time. We would like to audio-record this interview, and this will be typed up, read only by us in the research team and your name will be taken off the written version.

Consented to audio recording: **Yes / No** (circle)

[Ensure that the participant is happy to continue and has provided consent – ensure it is **INITIALED**]

<u>EACH-B</u>

1. What did you think of the EACH-B project when you first heard about it?
2. How much do you know about your child taking part in the EACH-B study as part of their science class?
3. What did your child think of EACH-B when they first heard about it?
4. How did you feel when you found out your child's school was in the control arm?

<u>Life with your adolescent</u>

5. What does your child's day generally look like in terms of what they eat and how active they are?
6. What sort of changes have you noticed in their diet and lifestyle in the last few months? (since baseline)
7. Have there been any big changes at school in the last few months that might relate to your child's health and wellbeing?

Many thanks for your time.
