# Supplementary material for: Mixed-methods process evaluation of the EACH-B intervention in UK secondary schools: Delivery fidelity, stakeholder responses and contextual influences
Source: BMJ Public Health. 2025 Oct 21;3(2):e002491. doi: 10.1136/bmjph-2024-002491 (PMC12551551; doi:10.1136/bmjph-2024-002491)
Supplement: online supplemental file 13 [file bmjph-3-2-s013.pdf]

Supplementary material document 13: Parent topic guide intervention schools

EACH-B process evaluation interviews: Semi-structured topic guide

INTRODUCTION

Hello, I'm *[insert name]* from the University of Southampton & I'll be interviewing you today. Before we get started, I'd just like to run through a few things with you. We want to know how the parents of children who have taken part in EACH-B have found the experience, and if you think there is anything we could change or improve on. I'm going to be asking you about how you have found the study and how you think the experience has been for your children. Our chat won't last for more than 20 minutes and you are free to leave at any time. We would like to audio-record this interview, and this will be typed up, read only by us in the research team and your name will be taken off the written version.

Consented to audio recording: **Yes / No** (circle)

[Ensure that the participant is happy to continue and has provided consent – ensure it is **INITIALED**]

<u>EACH-B</u>

1. How much do you know about your child taking part in the EACH-B study as part of their science class?
2. What did your child think of EACH-B when they first heard about it?
3. How did they feel about wearing the Geneactiv watch?
4. How well do you think your child has engaged with the EACH-B project as a whole?
5. What do you think they have enjoyed the most/least about it?

<u>LifeLab</u>

6. What did they think about the LifeLab activities/trip?
7. What did they choose as their health pledge?
8. How well do you think they have stuck to their pledge?

<u>App</u>

9. How much do you know about the mobile app they were given?
10. How often do you think they have been using the app since they downloaded it?
11. What do you think they like the most/least about the app?
12. How do you think taking part in EACH-B has influenced them and their daily life?
13. How much do you think the app has influenced them and their daily life?
14. Have there been any big changes at school in the last few months that might relate your child's to health and wellbeing?

<u>Parent App</u>

15. How much have you used the parent website?
16. What do you think about the parent website?
17. What do you like the most about the parent website?
18. How could we improve the parent website?

Many thanks for your time.
