# Supplementary material for: Mixed-methods process evaluation of the EACH-B intervention in UK secondary schools: Delivery fidelity, stakeholder responses and contextual influences
Source: BMJ Public Health. 2025 Oct 21;3(2):e002491. doi: 10.1136/bmjph-2024-002491 (PMC12551551; doi:10.1136/bmjph-2024-002491)
Supplement: online supplemental file 14 [file bmjph-3-2-s014.pdf]

Supplementary material document 14: Control school student interviews coding table

Code	Description	Illustrative quotes
Contextual factors<br>• Eating habits and preferences<br>• Parental involvement in food choice or preparation<br>• Sleeping habits<br>• Health campaigns or policies in school<br>• Seasonal changes<br>• Changes to school environment<br>• Impact of COVID-19<br>• Impact of food advertising aimed at YP<br>• Changes to diet or exercise as a result of taking part in the study<br>• Physical activity behaviours	Students described a range of eating habits and preferences. They say eating healthy is difficult – e.g. they like energy drinks even though they know they are bad for them. They eat a mixture of healthy and unhealthy foods at home, because parents have a say in what they eat. The food they eat at school is almost entirely unhealthy and this is impacted by availability, taste, cost and the freshness/appearance of the food. They want value for money and fruit at school is more expensive than a cookie. They would prefer the food at school to be healthier, but it has to be cheaper too.<br>They are aware of school/teacher desire to encourage healthy eating and exercise but can't name specific policies or campaigns.<br>Many enjoy physical activity and this is mostly in the form of organised team sports or clubs like swimming. There are more opportunities for PA outside of school. Some did less PA during COVID, but now that the pandemic is over many feel they are more active.<br>Some feel that compared to when they were younger (end of primary school/year 7) they are more active now because they are allowed to go out and meet up with their friends which often involved walking into town or to get public transport etc. They are also more likely to walk or cycle to school now rather than getting dropped off by parents. They are having more PSHE lessons now than when they were younger.<br>They also enjoy 'doing nothing' which means sitting down scrolling on their phone.<br>Some felt that wearing the GAs made them want to be more physically active whereas others said they didn't	<u>Eating habits and preferences</u><br>"I feel like for my diet, I wouldn't say I'm particularly healthy, but I wouldn't say I'm particularly unhealthy. I feel like I'm kind of a balance in the middle, 'cause I do… like I have orange juice every morning, I take vitamins, I have at least probably two or three portions of fruit and veg a day. Maybe like four with the orange juice, but then again I can't imagine life without crisps or chocolate, or sweets. I feel like… I just couldn't give that up, and like you can't take small amounts as well, you just have to… although that being said, my mum does try and help me sort of regulate how much I have. And sneak vegetables into my pasta or whatever."<br><br><u>Parental involvement in food choice or preparation</u><br>**"Do you help with cooking at home, or...?**<br>No.<br>**No. Your mum does it all, mostly, or...? Mum and dad?**<br>Usually my dad, but when my dad's away, my mum does it. But she's not... she doesn't do it very well, so I just eat beans on toast.<br>**So you prefer it when dad does it?**<br>Yeah."<br><br><u>Sleeping habits</u><br>"I know there's a lot of people, I certainly do, like pull a lot of all-nighters, like not intentionally, like you'll just be sat there and you're not looking at what the time is. And then suddenly, parent come in, 'yeah, you need to get up and go to school,' and I'm just sat there like, 'what? Huh?' And it's just like you have no concept of

Supplementary material document 14: Control school student interviews coding table

	think about it and just went about their usual week. Those who said the questionnaire made them think about their diets more than usual said that they didn't change their eating habits because of this.	time, (Audio obscured) time when you're using like a phone or something, or like you're just not doing anything."<br><br><u>Health campaigns or policies in school</u><br>"They do encourage us to, you know, do more activity, like the extra curriculum thing, so we were offered a lot of that, like cadets, clubs, all this other sort of stuff. Yeah. But then I, I do find it a bit counterproductive that some of the stuff in the canteen, if you had like more than one or two a day, I feel like you would have a heart attack."<br><br><u>Seasonal changes</u><br>"I think it depends on what sort of thing you do, 'cause if you do sports and that, it depends like when it is, 'cause you might play a sport where there's like a big season from, I don't know, October to... I don't know, like April. But it, it really depends on what you do, I think… Winter sports and stuff."<br><br><u>Changes to school environment</u><br>"Yeah, in the canteen it's cheaper to be unhealthy than healthy, I'd say…They changed it last year to be healthier, but like everything that's healthy has just gone up loads, so… it's still the same, pretty much."<br><br><u>Impact of COVID-19</u><br>"So it was actually coming out of lockdown, slightly mixed, yeah, really weird. We had groups, and then we had to wear masks….I mean, like I'd probably do more like exercise, because like football training wasn't back on then…"

Supplementary material document 14: Control school student interviews coding table

		<u>Impact of food advertising aimed at YP</u><br>"I've found this out recently, but the fast food adverts are only allowed on up un- like until nine o'clock until like whenever. But like the problem with that is, I stay up, so like my dad like go to like Tesco's, the advert comes on, and like 'oh, they can get this for like 50p, yay.'"<br><br><u>Changes to diet or exercise as a result of taking part in the study</u><br>"I think having the watch on pushed me a bit more to do more things, but then when I didn't have the watch I was a bit like, well it's gone now, I can sort of relax."<br><br>"Probably like realising what you, you actually yourself does, even, because you don't actually realise, you don't think about it on a day to day basis. Like how many crisps have I had today? How much exercise have I actually done today? It's just like sort of realising, oh actually, maybe I need to do more, or maybe I do enough."<br><br><u>Physical activity behaviours</u><br>"I do training twice a week for like an hour and a half each time. I think, I ride there a lot on my bike to get there. One of them's in a… like it's quite, it's quite far, it's a good few miles, so it's quite a lot of exercise. And we do lots of like running at the start of it, and fitness first aid, so yeah."
Engagement with data collection<br>• Quantity of wearing GAs<br>• Reasons for not wearing GAs<br>• Understanding how the GAs work<br>• Starting the GAs with a button press	Some students wore the GA for the whole week. Many took it off to shower, for sports, or just didn't wear it at all because it was uncomfortable.<br>Some were concerned about whether the GA was tracking them, but had felt reassured when they were	<u>Quantity of wearing GAs</u><br>"I think a lot of people just took it off anyway"<br>"I did not take it off once."<br><br><u>Reasons for not wearing GAs</u>

Supplementary material document 14: Control school student interviews coding table

	told this wasn't the case. Students who had to activate their GA by pressing the button had found this confusing and difficult.	"I had to take it off when I went to sleep, 'cause I have my hand under my pillow, so it was like rubbing on my wrist."<br><br><u>Understanding how the GAs work</u><br>"So I did have a look at it, and it's got everything from like an accelerometer so it like detects movement. And I heard that it can detect different bits, like whether you're scratching and stuff like that. And I think on the front it also had an ambient light sensor"<br><br><u>Starting the GAs with a button press</u><br>"I was scared. I feel like, I was tempted, I like pressed it a few times, and I was scared that like reset it."<br>"I think there was a bit of confusion at the start, from like actually turning it on and like putting it on and stuff. But then it was fine once we got, kind of got the hang of it."
Positive reactions to data collection<br>• Positive reactions to the questionnaire<br>• Positive reactions to wearing the GAs	Students said the GAs were uncomfortable at first and took a bit of getting used to but mostly they were fine to wear for the whole week. They found the idea that they were wearing a piece of important scientific equipment exciting.<br>They thought the questionnaires were easy to understand and fill in. They thought it was fun and cool to be part of real research.	<u>Positive reactions to the questionnaire</u><br>"It was quite fun, actually. 'Cause I'm weird (Yeah, I enjoyed it), I love like questionnaires and stuff. It was quite interesting, actually, 'cause it just... it really makes you think about the reality of how much you have in terms of like vegetables and naughties and stuff like that."<br><br><u>Positive reactions to wearing the GAs</u><br>"It was all right. Like it wasn't anything like crazy, it was something simple that anyone could really do. So it was something that you didn't have to worry about working your life around it, you could just put a watch on and call it a day."<br>"It just sort of felt like wearing a normal watch."

Supplementary material document 14: Control school student interviews coding table

Negative reactions to data collection<br>• Concerns about anonymity and data protection<br>• Negative reaction to the questionnaire<br>• Negative reactions to wearing GAs<br>• Not understanding relevance of the questionnaire	Some students had concerns about anonymity of the questionnaire, or found it too long or confusing.<br>Some students found the GAs very uncomfortable, or that it got in the way of the apple watch/fitness watch they already wore.	<u>Concerns about anonymity and data protection</u><br>"They might not want you to like monitor what they do on their like exercise wise, or how they get to school, whatever they do. They might not want it to be monitored… Like probably just be privacy. They don't want anyone else to know. It's like knowing a lot of people, it's... they are pretty confidential. They don't like sharing a lot of things."<br><br><u>Negative reaction to the questionnaire</u><br>"That's another thing, the questionnaire, it was very long…It was long, yeah…It just felt quite repetitive."<br>"Like it kept on going and going, there were so many questions."<br><br><u>Negative reaction to wearing GAs</u><br>"It did get annoying at sometimes, 'cause you could just be doing something and it… and it would just get in the way."<br>"A bit itchy in places, though."<br><br><u>Not understanding relevance of the questionnaire</u><br>"it was like asking you what your mental state is, or how your home life is, or something like that. Or like how you are in, on a daily... or what you feel, or whatever like. Why would they need to know that stuff?"
Suggestions for improvements to EACH-B trial<br>• Improvements to data collection sessions<br>• Suggestions for improvements to the questionnaire	Students felt the questionnaire should be shorter or split up into separate questionnaires filled in over a number of days. They felt it was quite repetitive. They felt some of the images didn't help to clarify the portion sizes (e.g. a bowl of crisps doesn't help them to decide what they had if their reference is a small bag or a large bag).	<u>Improvements to data collection sessions</u><br>"I mean, I was a bit confused of like what the watch actually did, because we didn't know if it checked our heart rate, we didn't know if it knew if we were walking, or doing whatever. We didn't know if it was about food, like it was a bit confusing, just like…It just

Supplementary material document 14: Control school student interviews coding table

	Students also would've wanted to know more about how the GAs worked and what exactly they tracked.	being there, like you don't know what's getting tracked…Yeah, it was very much, 'here's this watch, wear it for scien- like for scientific purposes... off you go,' sort of thing."<br><br><u>Suggestions for improvements to the questionnaire</u><br>"I think if they made the questionnaire maybe not as long, and maybe... I think some of it was a bit too personal as well. Just cut it down a bit, filling it in would've been better."
Views on health<br>• Changing opinions on importance of health<br>• Health is a low priority<br>• Health is important<br>• Importance of balance<br>• Mental health<br>• Sleeping habits<br>• Support from family makes being healthy easier<br>• They don't talk to their friends about health<br>• Understanding of what healthy food is<br>• Views on diet and health	Students think that health is important but also acknowledge that health is not a priority for all young people. When they say health is important, they mean a balance of healthy diet, exercise, sleep and mental health.<br>They know they have access to mental health support from school but don't think many students use these resources. Their parents support them to eat healthily. Sometimes healthier food is more expensive (ie. At school), sometimes unhealthy food is more expensive (e.g. in supermarkets). When thinking about physical health they think about weight and body size.	<u>Changing opinions on importance of health</u><br>"Maybe they can change the way the world looks at health, as in like figures around it, and that could kids really care about what they're doing in a day, or when we're on the phone. But that would probably show future generations that maybe active, health, and the way people look at teenagers. 'Cause I don't think teenagers have a very good picture in certain people's minds, it could change the way like people look at them and how they act, and how they could change the way they live and all this."<br><br><u>Health is a low priority</u><br>"**Do you think that teenagers feel like their health is important, if that makes sense?**<br>No…I think some people do but some people don't."<br><br><u>Health is important</u><br>"I don't know, just like… just to not be fat really, and like not be like overweight, like I wanna be like healthy and like be able to do things like exercise and things like that."

Supplementary material document 14: Control school student interviews coding table

		<u>Importance of balance</u><br>"If you're sleeping too much, and then that means you're not eating as much as you should, or you're not exercising as much as you should, that can obviously factor a lot of things. But the same if you eat too much, eat too little, if you exercise too much, and then you're not getting enough sleep, it all needs to be balanced out evenly, otherwise some points will just fall a bit short."<br><br><u>Mental health</u><br>"mental health could be a whole range of things, it could be from people saying stuff, it could be from people like... or from like having your like mixed thing, like should I eat that or should I eat that? Like will people say something if I wanna eat that?"<br><br><u>Sleeping habits</u><br>"Well I think I know I need sleep, I'm generally a very tired person, and I know, I know I'm not helping myself much by not sleeping some nights. But I think sleep, honestly, out of all them, is the lowest priority for myself."<br><br><u>Support from family makes being healthy easier</u><br>"Like my... my mum at one point made like a really big enforcement on like being healthier and things, so like they got... my mum got like a spin bike and stuff, and like started getting like healthier meals to cook."<br><br><u>They don't talk to their friends about health</u><br>"**Do you talk about diet, health, anything like that with your friends?**

Supplementary material document 14: Control school student interviews coding table

		Not really…No…No, not really, it's not something you talk about"<br><br>Understanding of what healthy food is<br>"I'm always like pressuring my mum and like helping her out to try and make like a, like a vegetarian and healthy meal as possible. Because my sister and my dad, they love meat. But my other sister, me and my mum, we always wanna to like go more plant based and stuff"<br><br>Views on diet and health<br>"Vegans, they never have any like proteins, and they... that's why they ?? bones….Or anorexic people."<br><br>"I don't know, just like… just to not be fat really, and like not be like overweight, like I wanna be like healthy and like be able to do things like exercise and things like that."
Views on research<br>• Happy to take part in research<br>• Parents happy that students taking part in research<br>• Thoughts on EACH-B study	Students were happy to be part of real research and thought it was cool and exciting. They liked the fact that they were helping out scientists with their work. Their parents also liked that they were taking part.<br>They understood that EACHB was about health but weren't 100% sure of the details<br>Students weren't aware that they were in the control arm of the study but they would've preferred to be in the intervention arm when they found out. They thought LifeLab sounded good.<br>Students were excited about the prospect of EACHB being able to make positive changes to the health of future young people.	Happy to take part in research<br>"It's quite cool to think that I could almost give to something that could help sort of generations to come in health and stuff like that.<br><br>Parents happy that students taking part in research<br>"I think both of my parents liked I was involved in something…That's very true."<br><br>Thoughts on EACH-B study<br>"Well it was a bit exciting taking part in something like that."<br>"**What did you understand about why we were doing that research, and what it was for?**

Supplementary material document 14: Control school student interviews coding table

		Not much, at all."
What the students remember about EACH-B<br>• Memories of the GAs<br>• Memories of the questionnaire<br>• Unsure of purpose of GAs	Even several months after their baseline, the students remembered wearing the GAs and filling in the questionnaire, although their memories of the questionnaire were mostly about how long it was. They remembered being confused about what the GAs were tracking (heartrate, steps, location etc.)	Memories of the GAs<br>"It wasn't very ?comfortable? 'cause it didn't get in the way of anything, unless it got caught on something, like the door handle, it wasn't... really, didn't really notice it was there."<br><br>Memories of the questionnaire<br>"It was pretty easy…If you know what your daily lifestyle is, then it would be a piece of cake."<br><br>Unsure of the purpose of GAs<br>"I mean, I was a bit confused of like what the watch actually did, because we didn't know if it checked our heart rate, we didn't know if it knew if we were walking, or doing whatever. We didn't know if it was about food, like it was a bit confusing"
