# Supplementary material for: Mixed-methods process evaluation of the EACH-B intervention in UK secondary schools: Delivery fidelity, stakeholder responses and contextual influences
Source: BMJ Public Health. 2025 Oct 21;3(2):e002491. doi: 10.1136/bmjph-2024-002491 (PMC12551551; doi:10.1136/bmjph-2024-002491)
Supplement: online supplemental file 15 [file bmjph-3-2-s015.pdf]

Supplementary material document 15: Control school teacher interviews coding table

Code	Description	Illustrative quotes
Contextual factors<br>• Changes to the size of science department in school<br>• Exercise is framed as fun rather than to look a certain way<br>• Food in school<br>• Having health related conversations with students<br>• Health campaigns don't work unless they are meaningful<br>• Health policies in school<br>• Impact of COVID-19<br>• Lack of money in schools<br>• Lack of teachers in schools generally<br>• Parents don't have time to read long emails<br>• Physical activity in school<br>• School links with local clubs<br>• Wanting to push science as a subject in schools	Teachers described health policies and initiatives in school such as 'Education for Life'/PSHE classes, Festival of Sport, Inter-house competitions, the morning mile (walk), sports clubs, ban on phones etc.<br><br>Some said that PA is pushed more than healthy eating in school - PE seems to happen about once a week and then there are lots of extracurricular clubs. Most learning about health goes on in science lessons or food technology.<br><br>Teachers felt that the food served in the school canteen didn't necessarily reflect what the students were taught about a healthy diet. They say it's better than it used to be years ago but the food is still petty unhealthy and unhealthy food is cheaper than healthy food.<br><br>Some science departments had increased in size but others had reduced due to staff absences and difficulties retaining staff since COVID. This was also accompanied	<u>Changes to the size of science department in school</u><br>"And then from September we will have two technicians and four science teachers altogether. And in the next… you know, by the end of year eleven we'll be fully staffed, probably we will all have doubled in size again, with the exception of the technicians, I think we'll probably have a maximum of two…So it is… yeah, we are expanding, and we're expanding rapidly."<br><br><u>Exercise is framed as fun rather than to look a certain way</u><br>"Which is why I'm like, 'no, we run for fun and we run together.' And that healthy lifestyle, doesn't matter what you look like, you, you know, it's important."<br><br><u>Food in school</u><br>"It is a lot of... it is still sort of pizzas, and like baguettes with bacon and sort of, often popular are things like... actually, the fruit, even the fruit juices, if you look at them, aren't particularly healthy in terms of... I think they've got ten plus grams of sugar in some of the sort of small bottles of drink."<br><br>"The hot food option is nine times… like I would say four out of five days a week is a balanced option…So we have like stir fries and, you know, fajitas, and there's always…You know, there… the hot food isn't… it can be burgers and chips and a hot dog and chips, fish and chips. It can be, I'm not gonna lie. But it's not always that way, there is, you know, there's a roast dinner."

Supplementary material document 15: Control school teacher interviews coding table

		<u>Having health related conversations with students</u><br>"Don't really know, I suppose probably quite… probably relatively often, but only on a very kind of light touch, so I'd say "Oh, what have you got for lunch? Oh, it's all beige, have you got anything green?'"… But not like an in depth conversation, I wouldn't say."<br><br><u>Health campaigns don't work unless they are meaningful</u><br>"I think the bugbear for me is, they keep – and for the kids – they keep having days. It's healthy eating day, it's national fruit and vegetable day. They don't care….they certainly have zero effect in my opinion."<br><br><u>Health policies in school</u><br>"We do things in morning registrations, like we have one morning a week that's like a wellbeing morning and it's, there's a choice of kind of different things you can do. Some are active, like we've got a walking route round the site."<br><br><u>Impact of COVID-19</u><br>"That term in particular is pretty bad for us for COVID, we had a lot of sickness, and obviously Rice's class, because [name] was off, the engagement from her class wasn't as high as I would've liked."<br><br><u>Lack of money in schools</u><br>"And also it's expensive, isn't it? That's the thing, and schools have got less and less money."<br><br><u>Lack of teachers in schools generally</u>

Supplementary material document 15: Control school teacher interviews coding table

		"I work four days a week, and I've had several emails saying 'will you do us an extra day, we've got no teachers, and there's no supply staff available, all the agencies are booked out.'"<br><br><u>Parents don't have time to read long emails</u><br>"You sent the standard letter that I just used, I think it was a bit buried in it. And I think... well certainly from my point of view with two secondary school children at home, I get in from work, there's dirty PE kit, there's full lunch boxes, there's 'I can't find my maths homework,' and you look at your emails and there's an email that's got an A4 of closed text, and you're like da-da-da-da, anything I need to do? No. Gone."<br><br><u>Physical activity in school</u><br>"There are sort of just the usual sort of sports teams, aren't there? There's a football team for every year group, and there's the girls' football, there's basketball and different sort of seasonal sports throughout the year. Sort of... sort of the standard (Yeah) like cricket and athletics in the summer. Rugby and football in the winter type extra-curriculars."<br><br><u>School links with local clubs</u><br>"Well quite often we get adverts for different clubs that… so again, doing the daily notices in the morning, quite often there'll be an advert for a different activity or club that, you know, they're looking for members and things. And I think last week there was one for the… I think there's a fire sta- like a small fire station down in the town, and they run a cadet's club in the evening, and that kind of thing."

Supplementary material document 15: Control school teacher interviews coding table

		Wanting to push science as a subject in schools<br>"And that's where we struggle, we need to motivate them, we need to… 'no, I do want to get my three, my two GCSEs in science.' It's not just maths and English, 'cause although we're core, well if they fail science it doesn't matter."
Consent process<br>• Teachers actively chased parental consent	Most teachers felt the consent process was easy and went smoothly. Some had had to chase parents for opt-in consent or demographics consent but said they didn't mind doing this.	Teachers actively chase parental consent<br>"So we spent like a, both of us spent like a free each ringing parents and like resending it… Explaining, I remember one parent saying, "There's so much data, why do you need more data?" You know?"
Difficulties teachers had in facilitating their students to take part in EACH-B<br>• Communication between teachers about the study<br>• Time needed to organise participation was a burden<br>• Timetabling difficulties can limit time dedicated to study	The main challenge teachers faced when organising their data collection session was lack of time. There is so much going on in schools and teachers are under incredible pressure because of staff absences, lack of resources/money, timetabling difficulties, looking after students' mental health etc.<br>Most teachers felt glad they had been in the control arm from a workload point of view and felt that had they been assigned to the intervention arm they may have struggled more.	Communication between teachers about the study<br>"Yeah, 'cause you gave me all the information I needed. It was quite difficult to take over, but that's only because......and it didn't leave anything anywhere. So it was like, 'um, okay...' So this is where you got some, you probably got some weird emails going, 'we don't actually know what's going on...'"<br><br>Time needed to organise participation was a burden<br>"there was only certain points where it got kind of like, you know, a little bit stressy. And that's mainly again, it's all logistic stuff, and trying to, making sure... 'cause you don't want something to go wrong, or something you haven't done, so it can't happen."<br><br>Timetabling difficulties can limit time dedicated to the study<br>"the tricky thing is the timetable, and pulling children out of classes, because obviously yes they were together, they were together in science, but that doesn't mean that they're also all together in French and Geography and History and all the rest of it. So it's

Supplementary material document 15: Control school teacher interviews coding table

		a bit of an inconvenience to other colleagues, when you say, 'oh, actually, those fifteen are gonna be missing, by the way. Next week, on Wednesday.'"
Engagement with data collection<br>• Appealing to teachers' passion for science and research<br>• Data collection off-putting for anxious students<br>• Lack of engagement with questionnaire to provide meaningful answers<br>• Organising the data collections was easy<br>• Parent reactions and consent rate<br>• Quantity of wearing GAs<br>• Reasons for not wearing GAs<br>• Student concerns about anonymity and privacy<br>• Students engaging with questionnaire more in year 9<br>• Teachers reminding students about the study<br>• Views on students' response to the questionnaire	Teachers said that student mental health was one reason some students may not have engaged with data collection sessions (due to anxiety).<br><br>They also thought that some of the students had not engaged well with the questionnaire because it was so long and because they lost interest.<br><br>They thought most of their students had worn the GAs but there were the odd few in every class who they suspected had taken them off as soon as they got home.<br><br>They thought that sometimes this may have been because the students had concerns about privacy (GAs tracking them, the study's use of their data etc.) but had most often been because they looked ugly or were uncomfortable.	<u>Appealing to teachers' passion for science and research</u><br>"teachers are very stressed, but I mean, we are all in the profession... or should all of us be in the profession, you know, to share our love of science and research. And maybe it is sort of naive of me to sort of think that people are still enthusiastic about research and about real experiments."<br><br><u>Data collection off-putting for anxious students</u><br>Yeah, and just you know, three or four slides that we can talk around, just so that they… they know what's gonna happen. And then maybe even like you could put some screenshots, so 'your questionnaire's gonna look like this.'…'And you're gonna wear some activity trackers, and they're gonna look like that.'...get them comfortable with what's gonna happen. Yeah, that makes sense…Yeah, I think that you might get less of the like panicky questions"<br><br><u>Lack of engagement with questionnaire to provide meaningful answers</u><br>"A little bit, but then they're like, 'well I don't know the answers,' and well I can't tell you the answer, you have... you know, this is you. And I think that probably towards the end, some of them are just going a bit, just going 'tick, tick, tick, tick, tick, tick.' Rather than really thinking about the questions. So I wonder, first of all, if it could be presented in a little bit more of a child friendly sort of way?"

Supplementary material document 15: Control school teacher interviews coding table

		<u>Organising the data collections was easy</u><br>“I thought the actual... the organised like rolling it out and getting the survey done, and all of that sort of stuff, I thought was really smooth, it was good.”<br><br><u>Parent reactions and consent rate</u><br>“They probably did read it, and just thinking, ‘oh, this is another information gathering, they’ve got enough data, I’m not gonna give any more data.’…And they just… I don’t know what they concocted, and they just blew it out of proportion, and I was trying to, trying to explain…”<br><br><u>Quantity of wearing GAs</u><br>“So I think that the students who said, “I will wear it for the full seven days,” but end up taking it off, as a class teacher you kind of know, majority of them probably did wear it for the full seven days. But I could probably tell you and pick out the students who probably would’ve said, “Oh yeah, I’ve wore it for the full seven days,” but sort of took it off as soon as they got home.”<br><br><u>Reasons for not wearing GAs</u><br>“I think they are ugly, let’s be honest, so yeah, I think some of the girls especially were a bit embarrassed about wearing them a little bit”<br><br><u>Student concerns about anonymity and privacy</u><br>“I think really the only question that sort of got asked afterwards, it wasn’t, you know, why are we doing this, what’s gonna be done with it? It is... is my name gonna be down on it? Can they track me? Is it GPS?... And again, as soon as I sort of said “no, it’s... it’s legitimate

Supplementary material document 15: Control school teacher interviews coding table

		research, it's all anonymised, like you're fine, you're safe..."<br><br>Students engaging with questionnaire more in year 9<br>"I think, having just done the follow up one, they engaged with it much better this year than last year…And as we were saying, I'm not sure if that's an age thing or if it's 'cause they were familiar with the questionnaire…I'm not too sure what the reason is for that, but definitely this year they were more engaged."<br><br>Teachers reminding students about the study<br>"So all, every day last week it's been on the daily, and I've been in three different tutor groups over the course of the week. And each time, when I've said EachB they've gone "what's that?" And then I've said, "do you remember the questionnaire and the watches, activity watches?" And they've gone, "oh yes, we remember."<br><br>Views on students' response to the questionnaire<br>"So they… they found it purposeful."
Memories of data collection<br>• Memories of the GAs<br>• Memories of the questionnaire<br>• Geneactivs are more memorable than the questionnaire<br>• Completing data collection on phones was difficult for students<br>• Having personal IT equipment made data collection easier<br>• Teachers reinforced students to wear GAs	Teachers could remember the basic premise of the data collection sessions (student reactions to wearing the GA and to the content of the questionnaire). They felt that students remembered wearing the GAs more than they remembered filling in the questionnaire.<br><br>In schools that provided iPads to each individual student, data collection was really easy but in other schools some students had to complete the questionnaire on their phones and this made it more difficult.	Memories of the GAs<br>"With the other group, we did have a couple sort of handed in, and slight refusals. But I think that was largely because their teacher wasn't fully around to explain it all."<br><br>Memories of the questionnaire<br>"And each time, when I've said EachB they've gone "what's that?" And then I've said, "do you remember the questionnaire and the watches, activity watches?" And they've gone, "oh yes, we remember."

Supplementary material document 15: Control school teacher interviews coding table

		<u>Geneactivs are more memorable than the questionnaire</u><br>"I was gonna say, no it's less about the questionnaire, I think they've done the questionnaire they just forget about it, but the watches they notice and they remember."<br><br><u>Completing data collection on phones was difficult for students</u><br>"**There were some that were using their phones.**<br>And it just wasn't having any of it."<br><br><u>Having personal IT equipment made data collection easier</u><br>"Yeah, we are in a nice privileged place in, at Oasis School as well, 'cause they all have iPads."<br><br><u>Teachers reinforced students to wear GAs</u><br>"I went to collect it, and then they were like… I was like, "what have you done with it?" "I literally just took it off to play football." I was like, "why did you take it off to play football, you're not supposed to."
Positive reactions to data collection<br>• Positive reactions to the questionnaire<br>• Positive reactions to wearing the GAs<br>• Positive views on data collection process	Teachers said some students felt cool wearing the GAs and that they were proud/excited to wear it because it showed they were part of a research study.<br><br>Most teachers found organising and delivering the data collection session straightforward.	<u>Positive reactions to the questionnaire</u><br>"And they felt like, 'let's get this done,' you know? And they were… some of them are really, really into it, "carry on, you know, we've only got ten minutes left, quickly..." And they had to finish it at home, three or four pupils…So they… they found it purposeful."<br><br><u>Positive reactions to wearing the GAs</u><br>"They remember the watches, that was a good thing, I did that quite a lot, going "guys, does anyone remember the watches?"…And a lot of them were like,

Supplementary material document 15: Control school teacher interviews coding table

		"oh yeah, we do remember that, that was actually quite cool..."…'Can you do it again?'"<br><br>Positive views on data collection process<br>"So yeah, no, I think it's as easy as it would ever be to give out watches to every kid and get them to do a questionnaire."
Negative reactions to data collection<br>• Concerns about privacy<br>• Negative reaction to questionnaire<br>• Negative reactions to wearing GAs<br>• Reluctance of students to be interviewed	The main negative points were about the questionnaire being too long and some students not understanding some of the questions, and about the GAs being uncomfortable or ugly, making some students reluctant to wear them	Concerns about privacy<br>"Somebody asked, "oh, is it gonna know where I am? Is it going to be following ?? my location?""<br><br>Negative reaction to questionnaire<br>"I think they started off really well, but I think that their concentration span went, I think it was too long…And I think it was quite wordy"<br><br>Negative reactions to wearing GAs<br>"Then there was like the, 'oh, it's so uncomfortable, I don't think I can bear it any longer.'"<br>"There was the couple in the session at the start that were just, 'no, I'm not wearing it.'"<br><br>Reluctance of students to be interviewed<br>"Most said yes. There's a few that were like, 'oh...' But that's 'cause they were worried that they'd be asked for an interview, so you turn round and go, 'well I'll just say no to the interview.'"
Suggestions for improvements<br>• Improvements to trial delivery or data collection<br>• Improving letters to parents	Teachers thought it would be a good idea to break up the questionnaire or shorten it if possible. They also wanted adaptations for SEN (special educational needs) or EAL (English as an additional language) students.	Improvements to trial delivery or data collection<br>"I think it would be really important for them to be shown, like it doesn't have to a PowerPoint presentation, but just for them really to understand that they are part of something that's huge, and that like this is really exciting, and they're doing real science.

Supplementary material document 15: Control school teacher interviews coding table

	Some teachers thought that it would be good for the EACH-B team to spend more time explaining the study to the students, the purpose of the study and to really hammer home how important it was.	I feel some of that was lost, and I think – given that we had the entire hour – I feel that there could've been time taken at the beginning to really explain to them, and get them excited for what they were about to take part in."<br><br><u>Improving letters to parents</u><br>"I think one, I think one for the opt out. 'Your child has been selected to take part in this study with the University of Southampton. Please see the attached document about it. If you don't wish your child to take part, click this link.' And then another one when they've done it: 'thank you very much for allowing your child to take part in this. We would like – as part of the study we'd like to share their demographic information, it will be kept anonymous, please click this link…Short and sweet, so you can scan it at the same time as cooking tea and all the rest of it."
Views of parents<br>• Difficulty obtaining parental consent for demographics<br>• Parents reluctance for child to take part in the study<br>• Teachers communication with parents	Some teachers had struggled to get parents to give consent for their child's demographics to be used. But mostly this process had gone smoothly. They felt able to reassure parents who had concerns.	<u>Difficulty obtaining parental consent for demographics</u><br>"The demographics thing, 'cause that's opt-in, that was a bit of a hassle, chasing them down to try and get up to the right number of people to include the demographics form."<br><br><u>Parents reluctance or child to take part in the study</u><br>"The one there that had a wobble and wouldn't, didn't... suddenly didn't want to take part in the... one of the rooms that I, I was in... which room? Anyway, the room I was in. She... her mum then emailed in to say, 'just to confirm she doesn't wanna take part.'"<br><br><u>Teachers communication with parents</u>

Supplementary material document 15: Control school teacher interviews coding table

		"No, I mean, I sent a message to them anyway..After the follow up, and going, 'look, just to let you know that the follow up session is happening, so if you have any issues or questions, let me know.'"
Views on health<br>• Mental health	Mental health was a big topic for teachers. In some schools the students had requested more support with their mental health and schools had provided this in the form of support groups/facilities/workshops. In some schools the teachers did not feel the students had taken advantage of these facilities. In others they teachers felt that mental health support was being accessed regularly by students. They felt that stress was a key factor in students' declining mental health.	<u>Mental health</u><br>"talk about mental wellbeing, there's a lot of the students were saying 'we want more help with our mental health,' you know, set up a load of workshops at lunchtime. Interestingly everyone said they wanted more input on that, but no one went to the workshops."<br><br>"the Trinity has been… it's a vital part now. Because we have students who find it difficult to cope, you know? Stresses and strains, after being you know, under lockdown. And then suddenly overwhelmed with the amount of stuff they have to do and catch up with."
Views on research<br>• Good for students to learn about the research process<br>• Happy to take part in research<br>• Thoughts about EACH-B study	Teachers said they would be keen to engage with future research and thought their students would be too. They saw the value of students learning about science and the research process by taking part in real research.<br><br>Teachers felt it would be a great opportunity to bring students to LifeLab and thought the students would engage and enjoy it. However some felt that organising the trip would add to their workload/burden, particularly since COVID had increased their stress and workload levels so much already. They felt that being in the control arm had made their lives easier.<br><br>In general, they felt that students had had a positive reaction to taking part in the study.	<u>Good for students to learn about the research process</u><br>"I think it's been quite interesting for the students to see research actually happening, 'cause again otherwise they wouldn't normally have access to that kind of experience."<br><br><u>Happy to take part in research</u><br>"All of the students, or most of the students were very happy and very excited to sort of take part in actual research."<br><br>"Generally really excited. They were, felt quite privileged that they'd been chosen, no reservations whatsoever. They, yeah, they seemed really, really pleased and excited."

Supplementary material document 15: Control school teacher interviews coding table

	Teachers generally understood the main aims of EACH-B.	Thoughts about EACH-B study<br>"I think for the students it, in terms of like the EACH-B and the LifeLab stuff, I can sort of... like you said, with the... the fact we were the... the... the arm that we were, it would've been nice if we were to get the kids the experience of going into LifeLab and those sort of things."
