# Supplementary material for: Mixed-methods process evaluation of the EACH-B intervention in UK secondary schools: Delivery fidelity, stakeholder responses and contextual influences
Source: BMJ Public Health. 2025 Oct 21;3(2):e002491. doi: 10.1136/bmjph-2024-002491 (PMC12551551; doi:10.1136/bmjph-2024-002491)
Supplement: online supplemental file 16 [file bmjph-3-2-s016.pdf]

Supplementary material document 16: Intervention schools (student and teacher interviews) coding table

EACH-B Process Evaluation – INTERVENTION Interviews Coding Summary (based on the MRC guidance)

MRC framework factor	(STUDENT) Description	(STUDENT) Codes	(TEACHER) Description	(TEACHER) Codes
**Implementation**				
**Implementation process** *How delivery is achieved, training, resources etc.* (teacher interviews – PD days, delivering the LL lessons in school, organising LL trips, receiving and using the flight cases)<br><br>*The structures, resources & mechanisms through which delivery is achieved*	Many students felt that in order to maintain their interest, engagement and motivation to achieve their pledges they needed support from teachers and parents. They thought it might be helpful if teachers set homework related to achieving your pledge, made it into a competition or did weekly check-ins. This suggests that despite the HCS training, teachers may not have been following up with their students' pledges or having conversations around supporting the students to achieve their goals.	• Support needed to maintain health pledges	Lots of teachers felt under pressure to deliver the LifeLab curriculum or flight cases and some felt they hadn't been able to do this as well as they would've liked due to time/resource constraints. Staff shortages, particularly throughout COVID, made this worse. Teachers were also unsure of how to best support their students to download and use the app because they felt they didn't know enough about the app.<br><br>Some teachers were particularly invested and enthusiastic about LifeLab and EACH-B which often meant they were more organised and motivated. These teachers generally had previous experience of LifeLab.<br><br>Others struggled with the workload in terms of organising the LifeLab trip, flight case lessons or data collection sessions. They felt they just didn't have	• Feeling of not doing justice to intervention<br>• Staff shortages in science departments<br>• Teachers previous knowledge of LifeLab/Teacher enthusiasm for EACHB and LifeLab<br>• Teachers unsure of how to support student with app<br>• Workload for teacher/Organising LifeLab trip/ Stress or difficulties of taking

Supplementary material document 16: Intervention schools (student and teacher interviews) coding table

			the time to give these tasks their full attention.<br><br>Teachers agreed with what the students told us regarding the health pledges. They felt that most students needed reminding about their health pledges and that perhaps having the pledge integrated into the app so that students would receive prompts or some sort of reward for achieving their health pledge would be good.	part and organising things<br>• Difficulty collected GAs back from students<br>• Support needed to maintain health pledge<br>• Communication with teachers
**Fidelity**<br>*Whether the intervention was delivered as intended – the consistency of what is implemented with what was planned* (teacher interviews – how many teachers attended PD day and why/why not, receiving and distributing app logins)	Some students missed out activities at Lifelab due to misunderstanding the instructions (e.g. blood pressure measurement) or, when using a flight case, if the teacher ran out of time during the lessons.	• Reasons for not wearing the GAs<br>• Difficulty doing blood pressure measurements at health circus<br>• Running out of time to do lifelab practicals	Teachers had mostly positive responses to the PD/HCS training and felt that in theory it was a great idea. Some schools were only able to send one teacher, due to illness or logistical constraints, and sometimes that teacher fed back to other teachers within the department. Most teachers when asked remembered attending either an online or in-person PD day.	• Introduction (to data collection session) provided by researcher<br>• Not completing questionnaire in time and trying to complete at home<br>• Engagement with HCS training/positives of

Supplementary material document 16: Intervention schools (student and teacher interviews) coding table

		in school (flight case)		PD day and HCS training<br>• App download
**Dose**<br>*The quantity of intervention implemented*	Many students who managed to download the app did not use it at all and those who did use it tended to use it a few times and then never again. Some classes ran out of time to complete all the LifeLab activities and experiments provided in the flight cases.	• Quantity of wearing the GAs<br>• Quantity of app use<br>• Running out of time to do lifelab practicals in school (flight case)<br>• Reasons for not using the app	Teachers generally felt that there was a very small number of students actually using the app. Some had had conversations with students who had said they just weren't interested "If I wanted to play Candy Crush, I'll play Candy Crush" and others hadn't heard the students talking about the app at all.<br><br>Most (if not all) teachers who took part during the time when flight cases were being used said they had run out of time to deliver all aspects of the lifelab lessons/flight case activities in school.<br><br>Teachers said that some of the scientists from 'meet the scientist' were better than others.	• Quantity of wearing the GAs<br>• Quantity of app use<br>• Difficulties with flight case/Not enough time for LifeLab stuff in school (flight case)/Number of modules taught<br>• Some 'meet the scientists' were better at speaking to young people than others

Supplementary material document 16: Intervention schools (student and teacher interviews) coding table

**Adaptation**<br>*Adaptations/alterations to make the intervention fit different contexts vs. changes that undermine intervention fidelity* (teacher interviews – teachers delivering LL programme independently/flight cases, PD training online)	Most students who took part in flight case activities had a positive experience (only complaint was running out of time to complete all activities) and weren't aware that they would usually have been on a trip to LifeLab.	• LifeLab at school (flight case)<br>• Positive reaction to LifeLab flight case activities	Teachers seemed to have tried their best to engage with the flight cases and deliver the lifelab curriculum to their students, but ultimately almost all teachers felt they didn't have time to adequately teach the lifelab lessons/flight case activities.<br><br>They felt that the virtual 'meet the scientist' activity from the flight case was not as good as the in-person session.<br><br>One teacher who attended lifelab at the Maths Science Learning Centre (MSLC; Highfield campus) felt that it was a shame it hadn't been at the hospital (he had previous experience of Lifelab at SGH) but that getting his students to Highfield was logistically easier given the location of the school.	• LifeLab at school (flight case)<br>• Online meet the scientist not as engaging<br>• Positive reactions to LifeLab at MSLC<br>• Consent process/benefits of opt-out consent
**Reach**<br>*Whether the intended audience comes into contact with the intervention and how* (details of student absence, certain students who were excluded or	Some students were unable to download the app because it crashed/didn't allow them to download it properly. Others received their log-ins a few weeks after initially being told about the app (for schools that took part during COVID) or lost	• Reasons for not downloading the app	Teachers talked about reasons for students not downloading the app and this was mostly due to the log-ins not being handed out, or being lost. By far the most common reason for teachers or students not being able to engage with lifelab modules was lack of time – teachers found that the lifelab	• Reasons for parents opting out<br>• Reasons for not downloading the app

Supplementary material document 16: Intervention schools (student and teacher interviews) coding table

students who were hesitant to take part in follow up, students without consent/opt-in consent challenges)	their log-in sheet so never got round to downloading it.	• Reasons for not engaging with lifelab modules<br>• Problems with playing some of the games	pre/post lessons and the flight case lessons were too long to realistically fit into their 1 hour lessons.<br><br>In terms of opportunities to use HCS, most teachers said it was hard to find time and that if those sorts of conversations did happen, they were likely to last 5 minutes or less, and happen in small groups rather than one-to-one. They said that as teachers they don't tend to have those conversations with students very often unless they're in the context of extracurricular activities which allow more time for talking (e.g. one teacher spoke about doing the Duke of Edinburgh award with a group of students and said they had longer conversations with the students during activities).<br><br>Overall teachers seemed enthusiastic about HCS but not all had been able to attend the training due to illness or not being able to take time away from school.	• Reasons for not engaging with lifelab modules<br>• Opportunities to use HCS with students<br>• Reasons for not attending PD day<br>• Students who didn't engage with data collection<br>• Few one-to-one chats for HCS/not enough time to use HCS in school

Supplementary material document 16: Intervention schools (student and teacher interviews) coding table

Mechanisms of Impact				
**Participant responses**<br>*How participants interact with the intervention*	Students were generally keen for their parents to be included in the intervention (via a parent app or website).<br><br>Some students struggled to set a meaningful health pledge or did not see the point of setting one, but overall they loved LifeLab. They particularly enjoyed and appreciated the opportunity to learn about their own bodies and health. Some students talked about still thinking about it (their health + what they had learned) after the LL day was over and re-considering their own choices.<br><br>There were a very small number of negative reactions to LifeLab complaining that some of the activities were too difficult or that it was too loud.<br><br>Those who used the app enjoyed it but it's missing a hook to get them coming back and continuing use over time. All of the app games were mentioned, with Gutsy being one of	• Reasons for not wearing the GAs/not using the app<br>• Reactions to parent website<br>• Reactions to possible parent app<br>• Thoughts on use of phone games<br>• Lack of enthusiasm for health pledge<br>• Positive reactions to the intervention	Teachers said that most students either weren't interested in the app because they were interested in other things on their phone, or just weren't interested in phone games at all. Some students felt the app was 'too young' for them. Most teachers felt the app hadn't been particularly popular amongst students.<br><br>Some teachers said they'd had parents who weren't happy or who were asking questions about why their child had taken part, but once the teacher had explained the study and the opt-out consent process to them they had been happy. The fact that the students' participation was anonymous helped to reassure the parents.<br><br>Teachers felt the students engaged with lifelab well and that the pre-post lessons or flight case lessons were fun. There was just too much for them to fit in.<br><br>Teachers said that whilst the students engaged with lifelab activities at the	• Discussions generated from questionnaire<br>• Reasons for not using the app/Reasons for deleting the app<br>• Positives of data being anonymised<br>• Parent engagement with information sheets/Confusion or negative reaction from parents<br>• Reactions to possible parent app<br>• Students participating and asking questions at LifeLab<br>• LifeLab pre and post lessons

Supplementary material document 16: Intervention schools (student and teacher interviews) coding table

	the most popular mentioned (it was familiar to them because of similarity to Candy Crush and other games of the same format).	• Negative reactions to Lifelab/the app<br>• Negative reactions to wearing the GAs<br>• App does not have a big impact<br>• Reasons for deleting the app<br>• Detective Doug/Bobbie Saves the World/Gutsy etc.<br>• Impact of intervention on diet and PA<br>• Engagement with app (most	time, they were quick to forget and that generally things that happen during lessons are not particularly memorable to students. They engaged with the pledge making exercise on the lifelab day but most teachers didn't think the students revisited their pledges after that day.<br><br>Most teachers really engaged with the HCS training and seemed to have genuinely tried to use those skills in school. Most felt that they hadn't been particularly successful in using them due to time/resource constraints, but could see the benefit and said they thought it was something they'd like to work on.	• Students have forgotten some of the LifeLab activities<br>• Negative reactions to wearing the GAs<br>• Negative reactions to LifeLab/Lack of enthusiasm for health pledge<br>• Negative reactions to the app/Thinking the app is too young for them<br>• Positive reactions to the questionnaire/wearing the GAs<br>• Positive reaction to LifeLab flight case activities/seeing knowledge increase/Lifelab

Supplementary material document 16: Intervention schools (student and teacher interviews) coding table

		popular games on the app)		• Positives of 'meet the scientist'<br>• Teachers seeing benefits of HCS<br>• Teachers seeing increased motivation for health from students
**Mediators**<br>*Intermediate processes which explain changes in outcomes (e.g. autonomous motivation, self-efficacy, social support etc.)*	Students get support from parents and some talk to their parents about food and health. Some have been influenced by their parents to eat healthily or try different diets e.g. vegan or vegetarian.<br><br>Students' views on health and research may have influenced their engagement with the intervention. Students felt that health is a priority for them, but when it gets too difficult to maintain or they feel like they're being forced to do something they lose motivation. There are lots of other things they need/want to focus on right now. Mental health and physical health go hand in hand but	• Motivation from parents to change diet<br>• Goal setting as a mechanism of the app's impact<br>• Views on research<br>• Views on health<br>• Parental support helpful in implementing	Some teachers said they thought that many students had struggled to remember their pledges and to act on them in the long term. They thought that some sort of check-in at regular intervals would help the students to remember their pledges and remain engaged with them. However one teacher also made the point that the most motivated/engaged students were less likely to see the benefit of making a pledge because they were already making healthy changes/setting goals for themselves, and the less engaged students would likely have been less motivated so also	• Students with siblings who have gone to LifeLab<br>• Remembering health pledges<br>• Difficult questions and wording in questionnaire/issues filling in questionnaire<br>• Views on health/trying to prioritise health in school

Supplementary material document 16: Intervention schools (student and teacher interviews) coding table

	they feel that mental health has to come first. If you're not happy then you don't care about your physical health and don't have the motivation/energy to look after yourself.<br><br>Students were generally excited by having the opportunity to take part in research. They felt it was important although they weren't 100% sure what the aim of EACH-B was or why they were being asked to do questionnaires and wear GAs. They mostly talked about the benefits to themselves as a direct result of taking part (rather than the wider benefits for other young people in the future) –e.g. Exciting going on a trip to LifeLab, getting out of normal lessons, learning about their health/bodies, being part of something.	health pledge/support needed to maintain health pledges<br>• Enjoyment of LifeLab practicals<br>• Changes in attitudes after LifeLab	may not have seen the benefits of making and sticking to a health pledge.<br><br>Teachers were enthusiastic about being involved in EACH-B and mostly said that their students had been excited to take part. In particular the students were enthusiastic about LifeLab and the lifelab activities. Most teachers said they would be keen to take part in future research although some said they would be happier to take a less involved role due to the stress/workload of having to organise everything (particularly during COVID)	• Views on research/EACH-B study
**Unintended pathways & consequences**<br>*"Side-effects" of the intervention, unanticipated or unintended*	Some students changed their behaviour during the week they were wearing the Geneactiv because they wanted to 'do well', whereas others continued as normal and didn't really think about it. Filling in the	• Possible impact of GAs on physical activity	Some teachers thought that wearing the GAs may have motivated the students to be more active during the week they were wearing them.	• Possible impact of GAs on physical activity

Supplementary material document 16: Intervention schools (student and teacher interviews) coding table

consequences of the intervention	questionnaires made some students think in more depth about the foods they usually eat.	• Possible impact of questionnaire on dietary behaviours	Some teachers planned to teach the lifelab module to other classes in the future.	• Planning to teach LifeLab module in future
**Context**				
**Contextual factors that affect the implementation/delivery and mechanisms of impact of the intervention** *E.g. values, context of collective attitudes (of the school/teachers/parents), peer groups, COVID-19, lockdowns*	Lots of students hadn't spoken to their parents about the study but those who had said their parents were happy they were taking part and had encouraged them to take part.<br><br>Those who had taken part in the study during COVID felt it was a shame they hadn't been able to go on the trip to LifeLab and that their experience of taking part in EACH-B in general would've been better if it wasn't during COVID. They felt that wearing the GAs was a bit pointless because during lockdown periods they weren't able to be very active.<br><br>Students mostly like playing games on their phones but the games they choose to play are more complex and	• Communication with parents<br>• Impact of COVID-19 (less physical activity happened during lockdowns, taking part in the study during COVID)<br>• Thoughts on use of phone games	Teachers found that COVID had had quite a significant effect on students' mental wellbeing. They also felt that it had been nice for the students to take part in EACHB because they hadn't been able to do anything particularly fun or interesting through the lockdowns. However, teachers also acknowledged the difficulties of delivering the trial during COVID, such as organising data collection sessions (one school posted GAs out to students' homes, several others received GAs from us and conducted the data collection sessions themselves without any members of the research team present) and lifelab trips/teaching flight case materials.	• Communication with parents<br>• Impact of COVID-19 (Impact on emotional wellbeing, less physical activity happened during lockdowns, social impact of COVID on YP, taking part in the study during COVID)<br>• Thoughts on use of phone games

Supplementary material document 16: Intervention schools (student and teacher interviews) coding table

	usually have reward elements or social functions which they enjoy.	• Context of app use		• Misinformation about topics covered by LifeLab from outside school<br>• Health pledge most effective for those with intrinsic motivation
**Causal mechanisms present within the context which could increase or sustain the effect of the intervention**<br>*E.g. school policies, healthy eating initiatives, school food environment, parental influence*	Support from parents and the school has helped young people to start to make healthier changes. For example, healthier food being introduced to the school canteen or family members encouraging students to try new, healthy meals.	• Changes in school environment<br>• Motivation from parents to change diet<br>• Parental support helpful in implementing health pledge	Changes to the school environment during the course of the study, which were often COVID-related. For example, teachers being off sick long-term. Also lack of healthy food available in school was a big issue that was talked about. Teachers said their school canteens served a mixture of healthy and unhealthy food but that realistically the students were drawn to the unhealthy foods, and there weren't any healthy eating policies or initiatives in place to discourage this.	• Changes in school environment<br>• Health promotion in school<br>• School food

Supplementary material document 16: Intervention schools (student and teacher interviews) coding table
