# Supplementary material for: Mixed-methods process evaluation of the EACH-B intervention in UK secondary schools: Delivery fidelity, stakeholder responses and contextual influences
Source: BMJ Public Health. 2025 Oct 21;3(2):e002491. doi: 10.1136/bmjph-2024-002491 (PMC12551551; doi:10.1136/bmjph-2024-002491)
Supplement: online supplemental file 17 [file bmjph-3-2-s017.pdf]

Supplementary material document 17: Parent interviews (intervention and control schools) coding table

EACH-B Process Evaluation – Parent Interviews Coding Summary (based on the MRC guidance)

MRC framework factor	Description	Codes
**Reach**<br>*Whether the intended audience comes into contact with the intervention and how*	Parents mostly knew almost nothing about EACH-B. Some hadn't heard of EACH-B or LifeLab and had no idea their adolescent had taken part (some of these were families where mum and dad don't live together, so adolescent may have been at dad's for the week they wore the GA). Most of parents' awareness of EACH-B was related to the GAs – if they had seen their adolescent wearing them that was what they remembered most. Awareness of the questionnaire and Lifelab was lower.<br><br>Some parents were sure there adolescent hadn't downloaded the app, either because they hadn't talked about it or because the parent controls the apps that the adolescent can download, and they hadn't seen any request to download the app.	• Parent knows their adolescent didn't download EACHB app<br>• Parents level of EACH-B knowledge
**Participant responses**<br>*How participants interact with the intervention*	Some parents felt their adolescent didn't really understand the purpose or importance of EACH-B or didn't care about taking part, whereas others thought that their adolescent enjoyed taking part and would've enjoyed the LifeLab visit. Others said that they had seemed 'fine' about it (no strong positive or negative feelings about wearing GAs). Some parents thought that younger YP may have engaged more, or that they are more likely to be influenced to change their behaviour by	• What adolescents think of EACH-B and research<br>• Parents views on taking part in research<br>• Parents views on how EACHB team communicate<br>• Parent views of the EACH-B app<br>• Parent views of parent website<br>• Parent views of LifeLab

	social media apps like TikTok and Snapchat, or if there were immediate tangible rewards.<br><br>Some parents had previous experience of taking part in research, or worked in research, or just had a personal interest in science. They were enthusiastic about them or their adolescent taking part in research and about the benefits of health research.<br><br>Some parents said that they get so many emails/letters from school that any letters about the research get lost. Others said they don't get too many and that email is the best way to contact them. Some said a posted letter to their house would have more chance of being read than a letter sent home with their child from school.<br><br>None of the parents were aware of the EACH-B webpage/website for parents. Some said they would look at it whereas other were reluctant to say they would engage with additional information about the study.<br><br>Some parents said they would use a parent/family app that was connected to their adolescent's app, but others said they wouldn't. Some didn't see what the benefit would be.<br><br>Some parents were aware of LifeLab but others had never heard of it. When it was explained to them most thought it sounded great and thought their adolescent would enjoy it.	• Parent views of GAs

Supplementary material document 17: Parent interviews (intervention and control schools) coding table

	Parent said they didn't think adolescents would be particularly motivated to wear GA. They weren't sure they would've understood the importance.	
**Context**		
**Contextual factors that affect the implementation/delivery and mechanisms of impact of the intervention** *E.g. values, context of collective attitudes (of the school/teachers/parents), peer groups, COVID-19, lockdowns*	Most parents we spoke to felt that their adolescent was fairly fit and healthy. Some said their adolescent went above and beyond to eat healthily and did lots of physical activity. Most physical activity was in the form of sports clubs, team sports, or activities that parents and YP did together. Many of the parents were also quite active and they felt they actively encouraged their YP to be active.<br><br>Parents talked about adolescent body image issues and the impact of social media on adolescent health.	• Parent views of adolescent health
**Causal mechanisms present within the context which could increase or sustain the effect of the intervention** *E.g. school policies, healthy eating initiatives, school food environment, parental influence*	Parents talked about how they deal with food and meals at home. Most said they encourage their adolescent to eat healthily and acknowledged that children learn eating behaviours from their parents. They described the different roles surrounding food preparation in the household and some said their adolescent liked to help them cook meals for the family, or that they encouraged their adolescent to help with cooking.<br><br>Most parents thought that healthy eating and physical activity was encouraged in school but that it may not be a	• Family food practices<br>• Importance of PA comes from family<br>• Parents thoughts on school environment

	huge focus. They said that perhaps PE wasn't pushed as much with older students (GCSE years) and that there could be more opportunities for physical activity in school. They said that healthy eating is probably not encouraged as much either.	
